# Person- and family-centred care in neonatology: a scoping review to identify existing definitions, models of care, and related categories of interventions

**DOI:** 10.7189/jogh.15.04263

**Published:** 2025-09-26

**Authors:** Andrea Togo, Ornella Lincetto, Jenny Bua, Ilaria Mariani, Marzia Lazzerini

**Affiliations:** 1 Institute for Maternal and Child Health, IRCCS “Burlo Garofolo”, WHO Collaborating Centre for Maternal and Child Health, Trieste, Italy; 2 Institute for Maternal and Child Health, IRCCS “Burlo Garofolo”, Neonatal Intensive Care Unit, Trieste, Italy; 3 Maternal Adolescent Reproductive and Child Health Care Centre, Faculty of Epidemiology and Population Health, London School of Hygiene & Tropical Medicine, London, UK

## Abstract

**Background:**

Person- and family-centred care in the field of neonatology (N&FCC) are promoted by many international agencies and scientific societies because of evidence-based benefits for infants, parents and health systems; however, being very broad and evolving concepts, they have not been uniformly defined in operational terms. We conducted a scoping review of literature relevant to N&FCC with the objectives of synthetising: 1) existing definitions; 2) models of care; 3) categories of interventions suggested by each model of care.

**Methods:**

We searched PubMed/MEDLINE, Embase, Web of Science, and Google Scholar for articles and/or grey literature published until 5 February 2024. For each objective, we considered articles and/or other documents, for any type of newborn.

**Results:**

The searches yielded 10 771 records. A total of 91 documents were deemed eligible for inclusion. We identified 40 relevant definitions and 28 different models of care of N&FCC. Both definitions and models of care were categorised in four macro-groups, based on their main focus: newborn and developmental care, parental participation to care, no separation between mother-baby, and miscellanea. Out of the 28 models of care, a total of 51 categories of interventions were identified, with a variable number (range 2–17) reported per each model. These were grouped in five macro-categories: individualised neonatal health care; organisation of care, human resources and policies; physical resources; health professionals (HPs) capacity strengthening and support; family empowerment and support. While most models included individualised neonatal care and family empowerment interventions, HPs were frequently neglected as beneficiaries of the intervention: only 11 models incorporated HPs capacity strengthening, only three proposed a wider support for HPs.

**Conclusions:**

We identified and synthetised numerous definitions, models, and categories of interventions, highlighting the need for further conceptualisation and standardisation around the concept of N&FCC, including the perspective from low-middle income countries’, and from both parents and staff involved in care.

Approximately 10% of newborns worldwide, translating to about 30 million infants annually, need varying levels of hospital care, ranging from prolonged duration of stay in neonatal intensive care units (NICUs) to simpler and/or shorter forms of hospital admissions [1].

Human neurological maturation begins in utero and continues into the postnatal period, representing a phase of high vulnerability [2].

A large body of evidence indicates that maternal separation and hospitalisation induce a significant stress in newborns, particularly those born prematurely or with medical conditions, as well as in their parents [3–6]. These infants may experience disruptions in developmental maturation due to their underlying health condition, maternal separation, exposure to noxious stimuli, and inappropriate handling, all of which can have potential long-lasting negative effects [7–11]. At the same time, the hospitalisation of a newborn places considerable emotional strain on parents, potentially impairing their mental health and well-being [12–21]. A recent study found that about three quarters of parents of infants in NICUs, in Italy, Brazil or Tanzania, were suffering from either depression, anxiety, or stress [22].

Based on this evolving knowledge, both person-centred and family-centred care have been proposed as beneficial approaches for the neonates and their families [23], however, clear working definitions of these concepts are still lacking.

Person-centred care aims at improving health outcomes of individual patients in everyday clinical practice, taking into account the patient’s needs, preferences, and rights [24,25]. Principles of person-centred care, as defined by the Person-Centered Care Committee, include: ethical commitment, holistic scope, relational focus, individualised care, cultural awareness and responsiveness, relationship and communication focus, people-centred collaborative diagnosis, systems of health care, education and research [26].

When applying person-centred care principles to the newborn to achieve newborn-centred care, it is important to emphasise that newborns are individuals with their specific needs (*e.g*. nutrition, sleep, pain control, need for loving relationship, *etc*.) in a delicate phase of development and with specific communication competencies even when born preterm or sick [27,28]. Over the past decades, growing evidence has highlighted the crucial role of interpreting neonatal cues to guide sensory and environmental adaptations and care practices, ultimately supporting newborns’ strengths and self-regulation capacities, and improving long-term outcomes. These aspects of care have often been incorporated in different models of newborn care, which have been identified with different terminology, including ‘individualised newborn care’ or ‘infant/newborn developmental care’ [29–33].

In parallel, family-centred care in the field of neonatology emphasises the importance of involving the family in the care of the newborn, in mutual collaboration with health care providers, with the objective of empowering parents, upholding their rights, increasing their practical skills on providing care, improving their well-being and at the same time promoting bonding and reducing noxious stimuli for the infant [34–37].

To ensure that the specific needs and rights of newborns are fully addressed (*e.g*. no separation from parents, adequate nutrition, pain prevention and management, cue-based care), newborns require intermediates to advocate and act on their behalf. These intermediaries are primarily the mothers, families and, when required for health-related needs, the health care professionals. The intermediaries need to be adequately empowered and equipped with the skills required to effectively fulfil this critical role [38–40]. Therefore, in the field of neonatology, person- and family-centred care are interconnected concepts that together support the health and development of neonates as active participants in the care that they receive, with mothers and families playing a central role in both advocating for and providing care.

However, as knowledge, resources and values have evolved, both newborn-centred care and family-centred care have been defined and operationally declined in different ways, often with frequent overlaps. In particular, the broad umbrella-term ‘family-centred care’ has included different approaches and heterogenous practices such as kangaroo-mother care (KMC), developmental/individualised care, newborn individualised developmental care and assessment programme (NIDCAP), family participatory care, family-integrated care (FICare), and couplet care, among others [41–44]. In some models of care, individualised newborn-centred care has been considered as an integral component alongside family-centred care, while in other models, it has been treated as a separate concept [42,45–47]. This variability in terminology and coexistence of different models of care, while contributing to advancements in newborn care, has also led to considerable confusion.

Despite operational differences in application, newborn- and family-centred models compared to the so called ‘conventional care’ have been proved to be beneficial in different settings, including both low- and middle-income countries (LMICs) and high-income countries (HICs), as well as in different care environments – such as maternity wards, special care baby units, and NICUs [21]. Several systematic reviews have shown that these approaches can effectively improve newborn health outcomes, reduce length of hospitalisation and rates of hospital re-admission, improve parental mental health outcomes and satisfaction, family relationships and bonding [4,48–52].

Efforts to link the two concepts in an operational way have been made previously, such as for ‘Infant and Family-Centred developmental care’, proposed by European Foundation for Care of Newborn Infants (EFCNI) and by Gravens Consensus Committee [30,53]. However, no prior review has systematically identified and synthetised definitions and models of person-centred care and family-centred care in neonatology, which we will call, in the context of this paper, for simplicity, with the term ‘newborn and family-centred care’ (N&FCC). To address this gap and provide a comprehensive framework we conducted a set of two scoping reviews with different and complementary aims. The first review aimed at identifying, listing, and ordering in macro-groups:

1) existing definitions relevant to N&FCC;

2) models of care described;

3) related categories of interventions proposed by each model of care.

A second review aimed at synthetising guiding principles and standards of care of N&FCC, and will be reported separately. The synthesis provided by these two reviews shall favour further collaboration among different stakeholders, as well as a more comprehensive evidence synthesis of the benefits of these interventions, wider implementation, and ultimately better quality care for newborns, their families, and health professionals.

## METHODS

### Study design

This scoping review adhered to the methodologies outlined by the Joanna Briggs Institute and utilised Arksey’s framework for scoping reviews, incorporating its latest updates [54–56]. The authors developed a review protocol before the beginning of the study screening process; this was not registered with PROSPERO, as PROSPERO does not accept protocols for scoping reviews. The PRISMA Extension for Scoping Reviews (PRISMA-ScR) was followed for reporting, the PRISMA ScR checklist is annexed as supplementary file (Table S2 in the **Online Supplementary Document**] [57].

### Study objectives

This review had the following three objectives:

1) identify definitions provided for N&FCC and synthetise them in macro-groups

2) identify models of care related to N&FCC, and synthetise them in macro-groups

3) identify categories of interventions proposed by each identified model of care and synthetise them in macro-categories of interventions relevant to N&FCC.

### Identifying relevant articles

We searched for all relevant studies published up to 5 February 2024 in four electronic databases, *i.e*. PubMed, EMBASE, Web of Science, and Google Scholar with no language restrictions. The search strategy was developed in four subsequent steps. First, we tabulated and compared keywords used in previous studies [43,44]. As a second step, the search strategy was optimised by adding additional keywords emerging from a first set of retrieved studies, and by improved use of Boolean operators. Third, the search strategy was tested, to assess whether it retrieved all relevant studies including those resulting from previous reviews [43,44]. The resulting final search strategy is shown in supplementary document (Appendix 1 in the **Online Supplementary Document**). In addition, we hand-searched reference lists of included studies. For PubMed, EMBASE, Web of Science we did not pose any search restrictions (*e.g*. language, publication date). For Google Scholar, the search was limited to the first 1000 results, in line with existing literature [58]. All records were imported into Endnote software and duplicates removed.

### Inclusion criteria and study selection

Criteria were specific for each objective; we considered for inclusion articles and/or other relevant documents (*e.g*. grey literature) related to N&FCC for any type of newborn including healthy newborns, from birth to 44 weeks of corrected age (*i.e*. even during and after discharge), in any neonatal care settings (*i.e*. not limited to NICU), and country. We excluded documents and articles written in languages not known to the authors (*i.e*. Chinese).

### Objective 1

We included all case/conceptual definitions relevant to N&FCC (*e.g*. KMC, developmental and individualised care, family participatory care, family-integrated care, couplet care, among others). For this specific objective we also considered for inclusion articles on concept analysis attempting to develop definitions relevant to N&FCC.

When multiple definitions were provided for the same model over time, we stored all available definitions, however we reported only the most recent one in our results. When the same definition of model of care was adapted with variations across different countries, we included all relevant retrieved definitions. We also included articles/studies even when the definition provided was not completely clear to the review authors.

### Objective 2

We included articles and/or other relevant documents describing N&FCC models of care, either if the authors self-defined a ‘model of care’, or when the model was aligned with the World Health Organization (WHO) definition of model of care which states that:

*A model of care is a conceptualization and operationalization of how services are delivered, including the processes of care, organisation of providers and management of services, supported by the identification of roles and responsibilities of different platforms and providers along the pathways of care* [59].

We included studies when the model of care implicitly reflected the general principles of N&FCC, even if the authors were not explicitly mentioning either family-centred care or newborn-centred care in their model of care (as for KMC).

We excluded:

– studies which did not name the model of care as a different model compared to previously included models;

– studies that focused specifically on a single intervention (for example: studies that supported breastfeeding without supporting developmental care; parental financial support without family-centred care; parental presence during procedures without family-centred care);

– when the involvement of the parents in the neonatal care was limited to less than one hour a day where/if authors did not describe other parental/infant interactions.

### Objective 3

We included all articles and/or other relevant documents identified for objective #2 and describing interventions for each model of care related to N&FCC.

If multiple articles or documents from the same author or research group were identified and described interventions for the same model, all relevant sources were included to ensure a comprehensive representation of the full range of interventions.

A sample cross-check of the first 20 study titles and abstracts was initially conducted by three authors (AT, OL, ML). Subsequently, one author (AT) screened all remaining titles and abstracts and retrieved the full texts of the relevant papers. To minimise the risk of selection bias, all potentially eligible studies were then assessed in full text and discussed by three authors (AT, OL, ML) to determine final inclusion. Any discrepancies were resolved through group discussion.

### Data extraction and synthesis

Data extraction forms were developed through an iterative process and pre-piloted on a sample of 20 studies and further optimised until considered satisfactory. Data extraction was performed by two authors in parallel (AT and OL) and further discussed with a third author (ML). To ensure alignment in data extraction and synthesis, regular (most often weekly) discussion sessions were held. Disagreements were resolved by consensus.

Data on the authors’ names, year of publication, country affiliation of the first author and their country income category (HICs *vs*. LMICs as per the World Bank categorisation [60]), were systematically collected and presented in tables and graphs. For WHO definitions/documents published by WHO, we considered the country of affiliation of the first author who originally described the N&FCC intervention.

Definitions and models of care were grouped based on similarities in focus (*e.g*. newborn, family, mother-infant dyad) and outcomes of interest (*e.g*. continuum of care after discharge, neurodevelopment outcomes), as identified through an in-depth analysis of the included studies.

For the categories of interventions, the initial division in macro-categories was guided by the categories established by Cochrane review on family centred care in the paediatric field conducted by Shields et al. in 2012 (environmental, policies, communication, educational and family support interventions) [36]. We further adapted this classification to the N&FCC context, by identifying the specific targets of the interventions (*e.g*. newborn, family, health care workers, organisation of care, physical resources). We also identified meso-categories, grouping similar sub-categories of interventions. This was an interactive process, until consensus was reached among all authors. We summarised findings in tables and text.

This being a scoping review, it did not aim at assessing risk of bias and effectiveness of different interventions.

## RESULTS

**Figure 1:**
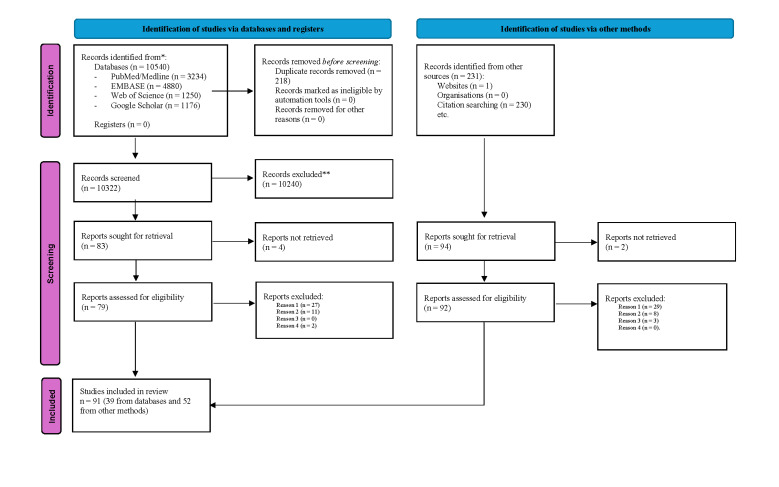
PRISMA Flowchart. Reason 1: Records did not name the model of care as a different model compared to previously existing models. Reason 2: Records focused specifically on a single intervention. Reason 3: Records excluded because the involvement of parents in neonatal care was limited to less than 1 hour/d or where/if authors did not describe other parental/infant interactions. Reason 4: Language barriers to authors’ interpretation (*i.e*. Chinese) [61].

The searches yielded 10 540 records from databases, and additional 231 records were identified from the citations and website searches (total of 10 771 records) (**Figure 1**). After screening and exclusion of duplicates, a total of 177 articles/documents were sought for retrieval (83 from databases and 94 from citation searching and websites) and 171 were assessed for inclusion (79 from databases and 92 from citation searching and websites). After the full text screening and paper discussion, a total of 91 records were overall considered eligible for inclusion (39 from databases and 52 from citation searching and websites), relevant to either objective 1, 2 or 3 (Appendix 2 in the **Online Supplementary Document**).

We identified 40 definitions, 28 distinct models and 51 categories of interventions related to N&FCC.

The definitions and models were classified into four groups: newborn developmental care, family participation to care, no separation between mother-baby, and miscellanea. Most originated from high-income countries and demonstrated a variable, and at times shifting, focus between the infant and the family. A recent trend toward integrating maternal and neonatal care was also observed.

The categories of intervention were grouped into five macro-categories based on their target: Individualised Neonatal Health Care; Organisation of Care, Human Resources, and Policies; Physical Resources; Health Professionals’ Capacity Strengthening and Support; and Family Empowerment and Support. However, no single model encompassed all five categories, with observed significant variability in scope and comprehensiveness.

### Definitions

A total of 40 documents reporting 40 definitions related to N&FCC were identified and reported in the supplementary document (Table S2in the **Online Supplementary Document**). The type of source documents for the definitions varied; 21 were derived from presentation of models of care, 12 were extracted from papers presenting recommendations or statements from working groups or professional bodies, six were identified in concept analyses, and one was obtained from a systematic review on family centred care. Only five documents were authored by researchers from LMICs.

Some definitions of the same model of care evolved over time. For example, NIDCAP gradually incorporated KMC as a key intervention [29,38]. Others adapted to different implementation settings, such as FICare which originated in Canada and was recently defined with variations in the UK [62–64].

We classified the definitions into four distinct groups (**Table 1**).

**Table 1:** Macro-groups of articles providing definitions related to N&FCC

Variables	GROUP 1	GROUP 2	GROUP 3	GROUP 4
Key focus	Newborn developmental care	Family participation to care	No separation between the mother-baby	Miscellanea: apply some of the N&FCC principles
Other aspects	Variable family involvement	Variable Newborn Developmental care	N&FCC not explicit but some N&FCC applied in delivering care for both – no separation, including in NICU and for mother with medical needs	
	McAlinden, B., et al. [65]. 'Baby Liberation' – Developing and implementing an individualised, developmentally-supportive care bundle to critically unwell infants in an Australian Paediatric Intensive Care Unit.	Institute for patient and family centered care [23]. What is Family-Centred Care. Patient and family centered care.	World Health Organization. [66] Kangaroo mother care: a transformative innovation in health care. Global position paper.	Kapito EM, et al. [67]. The H-HOPE behavioral intervention plus Kangaroo Mother Care increases mother-preterm infant responsivity in Malawi: a prospective cohort comparison.
	Pineda R, et al. [68]. Supporting and Enhancing NICU Sensory Experiences [SENSE], 2nd Edition: An Update on Developmentally Appropriate Interventions for Preterm Infants.	Pricoco R, et al. [69]. Impact of a family-centred clinical care programme on short-term outcomes of very low-birth weight infants.	Shuman CJ, et al. [70]. Integrating Neonatal Intensive Care Into a Family Birth Center: Describing the Integrated NICU (I-NIC].	Aita M, et al. [71]. Nurturing and quiet intervention (NeuroN-QI) on preterm infants' neurodevelopment and maternal stress and anxiety: A pilot randomised clinical trial protocol.
	Lisanti AJ, et al. [72]. Developmental Care for Hospitalized Infants with Complex Congenital Heart Disease: A Science Advisory from the American Heart Association.	Murphy M, et al. [73]. Effectiveness of Alberta Family Integrated Care on Neonatal Outcomes: A cluster randomised controlled trial.	Chellani H, et al. [74]. Mother-Newborn Care Unit (MNCU) Experience in India: A Paradigm Shift in Care of Small and Sick Newborns.	Schuetz Haemmerli N, et al. [75]. Interprofessional Collaboration in a New Model of Transitional Care for Families with Preterm Infants - The Health Care Professional's Perspective.
	Browne JV, et al. [30]. Gravens Consensus Committee on Infant and Family Centered Developmental Care. Executive summary: standards, competencies, and recommended best practices for infant- and family-centered developmental care in the intensive care unit.	Waddington C, et al. [76]. Family integrated care: Supporting parents as primary caregivers in the neonatal intensive care unit.	Klemming S, et al. [77]. Mother-Newborn Couplet Care from theory to practice to ensure zero separation for all newborns.	Mhango P, et al. [78]. Implementing the Family-Led Care model for preterm and low birth weight newborns in Malawi: Experience of health care workers.
	Aita, M, et al. [45]. The art of developmental care in the NICU: a concept analysis.	Hall SL, et al. [79]. The neonatal intensive parenting unit: an introduction.	de Salaberry J, et al. [80]. Journey to mother baby care: Implementation of a combined care/couplet model in a Level 2 neonatal intensive care unit.	Erdei C, et al. [81]. The Growth and Development Unit. A proposed approach for enhancing infant neurodevelopment and family-centered care in the Neonatal Intensive Care Unit.
	Macho P. [9]. Individualized Developmental Care in the NICU: A Concept Analysis.	Ramezani T. et al. [82]. Family-Centered Care in Neonatal Intensive Care Unit: A Concept Analysis.		Black, M. M., et al. [39]. The principles of Nurturing Care promote human capital and mitigate adversities from preconception through adolescence.
	Peterson, J. K, et al. [83]. Developmentally Supportive Care in Congenital Heart Disease: A Concept Analysis.	Asai H. [84]. Family-Centered Care in Perinatal and Pediatric Healthcare: A Concept Analysis.		Welch MG, et al. [85]. Family Nurture Intervention in the Neonatal Intensive Care Unit improves social-relatedness, attention, and neurodevelopment of preterm infants at 18 mo in a randomised controlled trial.
	Altimier L, et al. [86]. Newborn and Infant The Neonatal Integrative Developmental Care Model: Advanced Clinical Applications of the Seven Core Measures for Neuroprotective Family-centered.	American Academy of Pediatrics, Committee on Hospital Care [34]; Patient- and family-centered care and the pediatrician’s role.		Staniszewska S, et al. [87]. The POPPY study: developing a model of family-centred care for neonatal units.
	Craig J, et al. [88]. Recommendations for involving the family in developmental care of the NICU baby.	Mikkelsen G, et al. [89]. Family-centred care of children in hospital - a concept analysis.		
	Als H, et al. [29]. The Newborn Individualized Developmental Care and Assessment Program NIDCAP) with Kangaroo Mother Care (KMC): Comprehensive Care for Preterm Infants.	Davidson JE, et al. [90]. Guidelines for Family-Centered Care in the Neonatal, Pediatric, and Adult ICU.		
	Gibbins S, et al. [91]. The universe of developmental care: a new conceptual model for application in the neonatal intensive care unit.	Shelton T.L, et al. [92]. Family-centered Care for Children with Special Health Care Needs. Association for the Care of Children’s Health.		
	Liu WF, et al. [32]. The development of potentially better practices to support the neurodevelopment of infants in the NICU.	World Health Organization [93]. Standards for improving quality of care for small and sick newborns in health facilities.		
	Griffiths, N., et al. [47]. Individualised developmental care for babies and parents in the NICU: Evidence-based best practice guideline recommendations.	Shields L, et al. [94]. Family centred care: a review of qualitative studies.		
	EFCNI. [53]. European standards of care for Newborn Health: Infant-& family-centered development care.			

1. ‘Newborn Developmental Care’ (n = 14). This group included definitions primarily focused on the newborn’s neurological development, emphasising individual sensorial and communication needs, with variable participation of the family to support neonatal developmental and neurological outcomes [9,29,30,32,45,47,53,65,68,72,83,86,88,91].

2. ‘Family Participation to Care’ (n = 13). This group of definitions focused on the family, typically the mother or both parents, as key caregiver for the newborn. They emphasised the importance of supporting the family from different perspectives (*e.g*. skills development, social needs) to help them fulfilling their parental role, and mitigating the stress associated with the hospitalisation of their newborn. Some definitions in this group did not explicitly mention the newborn as main focus of their interventions [23,34,69,73,76,79,82,84,89,90,92–94].

3. ‘No Separation Between Mother-Baby’ (n = 5). This group focused on mother-infant dyad, minimising separation as a key strategy to enhance both neonatal and maternal outcomes [66,70,74,77,80].

4. ‘Miscellanea’ (n = 8). This group included definitions that did not fit within the other three categories or were less comprehensive. Some definitions in this group described N&FCC only in relation to specific moments of care – such as discharge – while others were developed within research projects [39,67,71,75,78,81,85,87].

A total 64 documents described 28 models, all with a different name. The 28 models of care, in the identification name provided by the authors, used a wide range of terminologies, with the most commonly being: ‘care’ (n = 13), ‘baby/infant/newborn’ (n = 12), ‘mother’ (n = 7), ‘family’ (n = 6), ‘integrated’ (n = 6), ‘developmental/developmentally’ (n = 4).

Similarly to the definitions, models could be classified into four distinct groups:

1. Newborn Developmental Care (n = 6). In the first group, models focused on individualised care, with health care providers responding to infant cues and adjusting the sensory environment to prioritise the newborn’s needs. While parents were often considered active participants, this was not universally the case [29,65,68,72,86,95].

2. Family Participation to Care (n = 7). The second group included models that emphasised the family’s role as the primary caregiver, incorporating supporting services and strategies to enable them to fulfil this role. In these models, the neonate was not always the primary focus but rather a beneficiary of the interventions aimed at supporting the family [63,64,69,73,76,79,96].

3. No Separation Between Mother-Baby (n = 6). The third group centred on the mother-infant dyad. The models in this group fundamentally reshaped both maternal and neonatal services by streamlining processes and integrating parental participation with responsiveness to neonatal cues. This approach ultimately led to the unification of maternal and neonatal care within a single service [66,70,74,77,80,97].

4. Miscellanea (n = 9). The last group included models that did not fit exclusively into one of the previous categories, or addressed specific aspects of care, such a discharge planning or palliative care. Some were developed as part of research projects [67,71,75,78,81,87,98–100]. (**Table 2**).

**Table 2:** Macro-groups of identified models of care related to N&FCC

	GROUP 1	GROUP 2	GROUP 3	GROUP 4
Key focus	Newborn Developmental care	Family Participation to Care	No separation between mother-baby	Miscellanea: apply some of the N&FCC principles
Other aspects	Variable family involvement	Variable Newborn and Developmental care	N&FCC not explicit but some N&FCC applied in delivering care for both – no separation, including in NICU and for mother with medical needs	
	McAlinden, B, et al. [65]. 'Baby Liberation' – Developing and implementing an individualised, developmentally-supportive care bundle to critically unwell infants in an Australian Paediatric Intensive Care Unit.	Pricoco R, et al. [69]. Impact of a family-centred clinical care programme on short-term outcomes of very low-birth weight infants.	World Health Organization. [66]. Kangaroo mother care: a transformative innovation in health care. Global position paper.	Kapito EM, et al. [67]. The H-HOPE behavioral intervention plus Kangaroo Mother Care increases mother-preterm infant responsivity in Malawi: a prospective cohort comparison.
	Pineda R, et al. [68]. Supporting and Enhancing NICU Sensory Experiences (SENSE), 2nd Edition: An Update on Developmentally Appropriate Interventions for Preterm Infants.	Murphy M, et al. [73]. Effectiveness of Alberta Family-Integrated Care on Neonatal Outcomes: A Cluster Randomized Controlled Trial.	Shuman CJ, et al. [70]. Integrating Neonatal Intensive Care into a Family Birth Center: Describing the Integrated NICU (I-NIC).	Czynski AJ, et al. [98]. The Mother Baby Comfort Care Pathway: The Development of a Rooming-In-Based Perinatal Palliative Care Program.
	Lisanti AJ, et al. [72]. Developmental Care for Hospitalized Infants with Complex Congenital Heart Disease: A Science Advisory From the American Heart Association.	Banerjee J, et al. [64]. Improving infant outcomes through implementation of a family integrated care bundle including a parent supporting mobile application.	Chellani H, et al. [74]. Mother-Newborn Care Unit (MNCU) Experience in India: A Paradigm Shift in Care of Small and Sick Newborns.	Schuetz Haemmerli N, et al. [75]. Interprofessional Collaboration in a New Model of Transitional Care for Families with Preterm Infants - The Health Care Professional's Perspective.
	Maria A, et al. [95]. Nurturing Beyond the Womb – Early Intervention Practices in Newborn Care Unit.	Patel N, et al. [63]. Family Integrated Care: changing the culture in the neonatal unit.	Klemming S, et al. [77]. Mother-Newborn Couplet Care from theory to practice to ensure zero separation for all newborns.	Aita M, et al. [71]. Nurturing and quiet intervention (NeuroN-QI) on preterm infants' neurodevelopment and maternal stress and anxiety: A pilot randomized clinical trial protocol.
	Altimier L, et al. [86]. The Neonatal Integrative Developmental Care Model: Advanced Clinical Applications of the Seven Core Measures for Neuroprotective Family-centered Developmental Care.	Hall SL, et al. [79]. The neonatal intensive parenting unit: an introduction.	de Salaberry J, et al. [80]. Journey to mother baby care: Implementation of a combined care/couplet model in a Level 2 neonatal intensive care unit.	Mhango P, et al. [78]. Implementing the Family-Led Care model for preterm and low birth weight newborns in Malawi: Experience of health care workers.
	Als H, B. et al. [29]. The Newborn Individualized Developmental Care and Assessment Program (NIDCAP) with Kangaroo Mother Care (KMC): Comprehensive Care for Preterm Infants.	Landsem IP, et al. [96]. Early intervention programme reduces stress in parents of preterms during childhood, a randomised controlled trial.	Levin A. [97]. The Mother-Infant unit at Tallinn Children's Hospital, Estonia: a truly baby-friendly unit.	Erdei C, et al. [81]. The Growth and Development Unit. A proposed approach for enhancing infant neurodevelopment and family-centered care in the Neonatal Intensive Care Unit.
		Waddington C, et al. [76]. Family integrated care: Supporting parents as primary caregivers in the neonatal intensive care unit.		Welch MG, et al. [99]. Family nurture intervention (FNI): methods and treatment protocol of a randomised controlled trial in the NICU.
				Staniszewska S, et al. [87]. The POPPY study: developing a model of family-centred care for neonatal units.
				Melnyk BM, et al. [100]. Reducing hospital expenditures with the COPE (Creating Opportunities for Parent Empowerment) programme for parents and premature infants: an analysis of direct health care neonatal intensive care unit costs and savings.

For the 21 models of care that provided a definition, there was alignment between the classification of definitions and the classifications of models.

**Figure 2:**
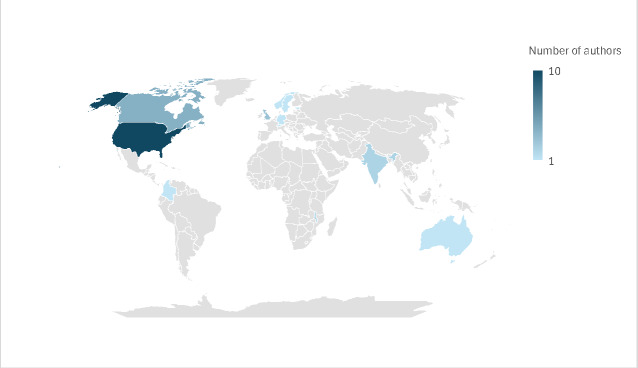
Country of origin of the first author of the identified model of care. In dark blue the country with the most first authors of model of care – in light blue the country with the least authors of models. In grey countries without any authors identified.

The first authors of the majority of models (22 / 28 = 82%) were from HICs, mainly from North America and Western Europe, with only six authors originating from LMICs, specifically from Colombia, Estonia, India and Malawi **(****Figure 2**) [67,78,95,97,101,102]. Notably, the two models from Malawi were developed through North-South collaborations [67,78].

**Figure 3:**
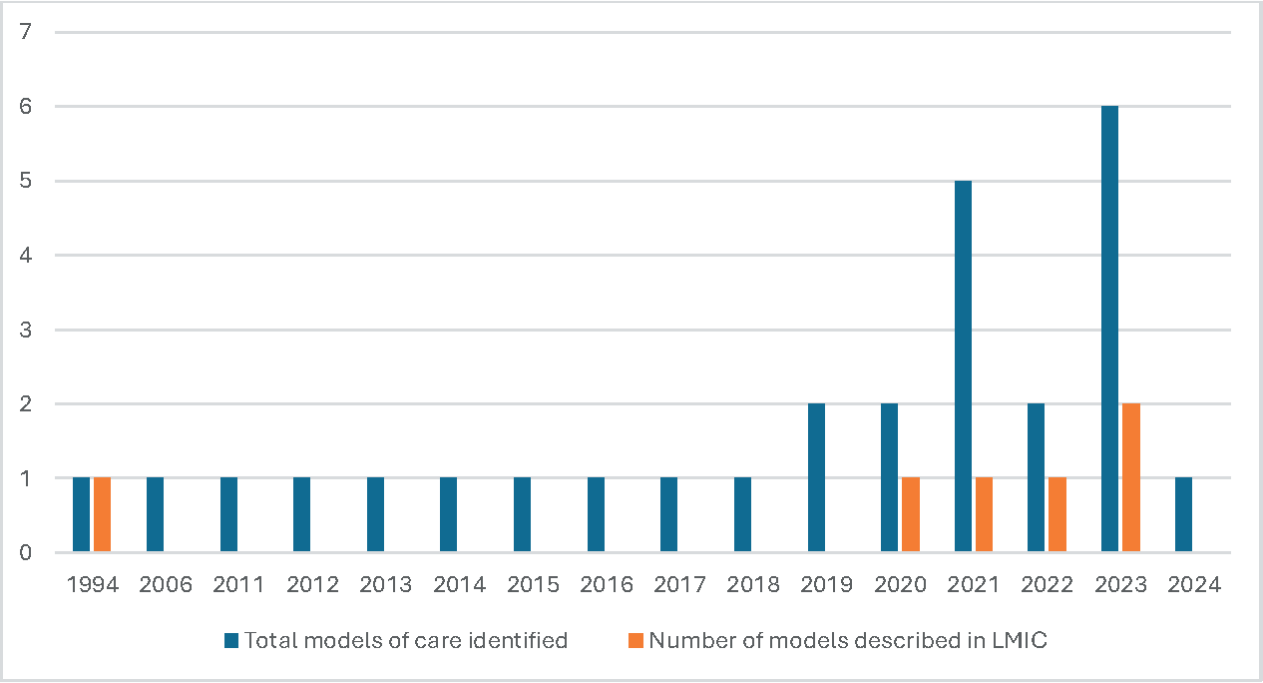
Models of care, by publication year and setting. y-axis reports absolute frequency. LMIC – low middle income countries.

Over time the numbers of publications describing new and updated models of care related to N&FCC increased, with 23 models (82%) published in the last 10 years compared to five models in the previous 10-year period (**Figure 3**). Notably, the types of models varied over time, with a growing trend towards integrating maternal care within family-centred models. This shift was evident in the increasing number of studies retrieved on KMC and mother-newborn couplet care (five models published in the last five years). These models emphasise active involvement of the mother, and at times of other family members, in the care of the infant, supporting the mother-infant dyad with minimisation of the separation of the baby from the mother and the family [66,70,74,80,103].

Regarding the extension and depth of change proposed, models differed widely. Some models implied significant changes in the organisation of care and in the physical structures to adapt to the neonatal needs, and to allow parental participation to care, as well as collaboration with health care workers. Examples of these models included: KMC, FICare, NIDCAP, Neonatal Integrative Developmental Care Model, Mother-newborn units, Growth and developing unit amongst others [29,76,81,86]. Other models of care proposed more focused interventions, either restricted to a specific group of patients (*i.e*. palliative care patients) or limited in time (just few hours a day), with variable involvement of parents, without substantial modification of conventional neonatal organisation of care and health care workers practices. Examples of these models included: The Mother-Baby Comfort Care Pathway, Creating Opportunities for Parent Empowerment (COPE), Mother Infant Transition Program (MITP) and Family Nurture Intervention [96,98–100].

Models also differed significantly on the timing of application: there were models in the ‘No Separation Between Mother-Baby’ (**Table 2**) that focused on the immediate postpartum period and continued throughout hospital admission, while others focused on the transition from the hospital to home and/or applying interventions of N&FCC in the community.

### Categories of intervention

Out of the 28 models of care identified, a total of 51 categories interventions were identified, with a variable number of them reported per each model (range 2 to 17). Quality of reporting was heterogenous, with some models lacking a clear description of the implementation practices, and others providing more detailed description, allowing for a better understanding of the characteristics of the proposed interventions.

The categories of interventions that had similar targets were grouped in five macro-categories, further divided into 13 meso-categories, which described the different strategies to reach their target: Individualised Neonatal Health Care (n = 5 meso-categories); Organisation of Care and Human Resources, Policies (n = 2 meso-categories); Physical Resources (n = 2 meso-categories); Health Professionals Capacity Strengthening and Support (n = 2 meso-categories); Family Empowerment and Support (n = 2 meso-categories) (**Table 3**). The full list of the identified categories of interventions of each model is available in the supplementary document (Appendix 3 in the **Online Supplementary Document**).

**Table 3:** Categories of intervention

Macro-category	Meso-category	List of categories of interventions
Individualised neonatal health care (promoting newborn and development)	Kangaroo mother care	Skin-to-skin as early and as long as possible
		Early and exclusive breastfeeding
	Sensory adaptation	Modification of external stimuli
		Detailed observation of infant behaviour
		Supportive positioning, gentle handing, massage
		Tactile interventions
		Vestibular interventions
		Olfactory and gustatory interventions
		Auditory interventions (including maternal voice)
		Kinaesthetic interventions
		Visual interventions
		Skin Care
	Pain management	Pain, sedation and withdrawal assessment, prevention and management, including procedural sedation
	Sleep protection	Individualised sleep strategies
		Cycled lights to support nocturnal sleep and facilitate development of circadian rhythm
	Nutrition	Individualised nutrition strategies
Organisation of care, human resources, and policies	Organisation of care and human resources	Prenatal consultations
		Integrated maternal and newborn care
		Interdisciplinary management teams and care practices
		Individualised nursing care activities
		Availability of mental health professionals, care strategies and policies
		Availability of specialised nurses in N&FCC and case managers
		Availability of lactation consultants
		Palliative and bereavement care
		Quality Improvement practices
		Partnering with families in policies development
		Strengthening referral system and follow-up care
		Community sensitisation and engagement
	Policies, guidelines and protocols	Discharge planning and post-discharge management (including early discharge)
		Establishment of guiding principles, standards of care, operating procedures and process description
		Policies for rooming-in and 24 hour access for parents
Physical resources	Environment improvement/redesign	Environmental changes focused on the infant
		Facilities specifically for families
		Facilities specifically for staff
	Equipment	Equipment adaptation for both newborn and mother/caregivers
		Equipment for staff
Health professionals’ capacity strengthening and support	Capacity strengthening	Structured educational programmes, including peer-to-peer education
		On-site capacity strengthening (*e.g*. mentorship, supportive supervision)
	Support	Structured programmes (*e.g*. for emotional support)
Family empowerment and support	Empowerment	Introduction to unit, staff, policies, patterns and routing
		Parental engagement as primary caregivers
		Competency programmes
		Peer-to-peer
		Parent-friendly information material
	Support	Mental health support
		Structured family support
		Respectful communication
		Social Worker support
		Financial support
		Post-discharge support from health visitor
		Community support and support groups

When the 28 models were analysed individually, none incorporated all five macro categories within a single model. Eight models covered four macro-categories [29,64,69,76,78,79,81,95], seven addressed three macro-categories [68,75,77,80,101,104,105], eleven addressed two macro-categories [63,65–67,70,87,96–99,106], and two models addressed only one macro-category [71,73].

Most models included family empowerment and support interventions (n = 23). In contrast, fewer models incorporated capacity strengthening interventions for health care workers (n = 11) [29,63,64,68,69,76–80,86], and only three addressed staff support, primarily emotional support [29,64,79].

## DISCUSSION

This review identified a wide range of definitions (n = 40) and models of care (n = 28) for N&FCC, which could be divided into four macro-groups, either more focused on neonatal and developmental care, or on parental participation to care, or the mother-infant dyad with zero separation, or on specific moments of care (*e.g*. at discharge, palliative care). Moreover, a high number (n = 51) of categories of intervention relevant to N&FCC were identified, pertaining to five macro-categories, directly targeting either the newborn, the family, the organisation of care, the physical resources or health professionals.

The heterogenicity and multitude of definitions, models of care and categories of interventions are not surprising, reflecting the complexity of N&FCC, its evolution over time and across different settings/groups and organisations, with each approach highlighting valuable aspects of this multifaceted concept. Most probably, the concept of N&FCC will continue to evolve in the future, as expected for any broad concept which includes both a technical component and a human rights-based approach.

Results of this review underscore an increased interest of the topic of N&FCC, over the most recent years, and a growing global movement to advance the knowledge and implementation of N&FCC. This progress has been driven by collaborative efforts from policymakers, health care workers, and parental associations, resulting in improvements in care practices.

However, this review also highlighted some fundamental gaps. First, the existence of many different definitions and models of care related to N&FCC may translate in a redundancy of programmes competing with each other, hampering wider implementation.

Second, none of the 28 identified models incorporated all five macro-categories of interventions, with most focusing only on some of the aspects, and neglecting either the family, or the newborn, or the staff. Similarly, across different models of care, there was high variability on the level of continuity of care across services. Although findings of this review reveal a recent shift toward an increase participation of the family as a whole (not just the mother) in neonatal care, and an increased integration across different services (maternal and newborn; hospital and community), still these aspects are not captured in all proposed models.

Third, the majority of definitions and models of care originated from HICs, underscoring a significant gap in research, evidence generation, and implementation from LMICs. It may be possible that some existing models of N&FCC currently implemented in LMICs are just not reported, rather than not existing. This imbalance highlights the need for more inclusive and geographically diverse research and efforts to bridge this gap and enhance the global relevance of N&FCC initiatives. While approaches to N&FCC need to be context-specific to be sustainable, examples such as the successful adaptation of FICare in China, the implementation of newborn and developmental care in India, and the long-standing practice of KMC in Northern Europe, demonstrate the potential for cross-cultural adaptation and implementation of various models and interventions across diverse countries [95,103,107,108]. As LMICs move towards a progressive expansion of neonatal services, this review could inspire investments in the uptake of N&FCC models of care that take into full consideration the needs of both newborns and their families with the perspective of better outcomes for the next generation of high-risk newborns and their caretakers.

Fourth, despite their fundamental role in delivering N&FCC care, health care professionals were often overlooked as direct beneficiaries of interventions. This highlights a significant gap in addressing health professionals’ needs, particularly in the stressful environment of NICUs.

The above cited gaps call for wider implementation as well as more research. In terms of research, first, to enhance N&FCC, there is a need for a comprehensive model of care that includes all key aspects, including possible collaboration across services, and all macro-categories of intervention, and clearly link them to underlying principles. Obviously, achieving wider implementation requires physical resources (*e.g*. economical, human), but also a global effort in better conceptualise the concept, named it in a more homogenous way, comprehensively list categories of interventions, and link them to underlying principles and standards of care. This will also allow better advocating and resource mobilisation.

Further implementation research is needed to explore and document the economic and cultural sustainability of different implementation approaches, both in HICs and in LMICs. Significant variation in N&FCC implementation across settings with different resources is not surprising but should not stop wider implementation. Newborn and family-centred care requires major changes in the organisation of the health care system, as well as consistent communication and collaboration between services, which can be challenging in siloed health systems and resources-limited settings. Further research can help identify what is feasible in each setting and the appropriate timeframe for implementation.

A wider implementation of N&FCC could benefit of lessons learned with the KMC as a model of care, which has a proven track record of over four decades of success history [109,110]. Effective implementation of KMC requires comprehensive approach, including family empowerment and support, health care staff capacity building, neonatal unit reorganisation and change in environment. However, this approach improves newborn outcomes, mitigates staffing shortages, reduces health care workload, strengthens parental skills and promotes the continuity of care, especially where socio-economic support services are lacking [111,112].

It is not surprising that KMC has evolved over time to be recommended for very small infants from an early stage [113]. The broader concept of N&FCC may follow a similar trajectory. Our review identified KMC as both a distinct model of care and an intervention integrated within various models of care, particularly those that emphasise the role, needs and rights of both infants and parents. Consequently, we classified KMC into different categories based on the study objectives: as a definition, as a model, and as a meso-category within individualised neonatal health care.

Finally, for a wider and more effective implementation, standards of care, along with globally recognised indicators, monitoring and evaluation frameworks, will be critical. These elements will allow for systematic assessment of progress in N&FCC implementation and the evaluation of its effectiveness in improving outcomes.

We acknowledge, as a limitation of this review, the challenges posed by the heterogeneity of articles describing definitions – some with an operative focus while others more conceptual – and the lack, in some cases, of a detailed description regarding models of care and their related categories of interventions. As a result, some subjective interpretation was necessary when categorising items that were not fully described by the authors. Further collaborative efforts may enhance the description of each model of care, and, most importantly, support the conceptualisation a comprehensive model of care which can benefit newborns, their families, health care professionals and the entire health system.

Similarly, the variability observed across models of care in the quality of reporting may have influenced the number of intervention categories identified. Publication bias, particularly for reports from LMICs, may also have affected our findings. In addition, although a sample cross-check of the first 20 results was conducted by three authors to mitigate potential bias, the remainder of the screening was performed by a single author, which may have introduced a risk of selection bias.

Another potential limitation of this review is the decision to include only the most recent definition of each identified model, which could bias the review by excluding earlier or less current interpretations. However, this approach ensures the inclusion of the most up-to-date definitions. Lastly, a limitation of our review is that categories of interventions were derived from articles describing models of care. A previous systematic review comprehensively synthetised all possible interventions related to family centred care for newborns evaluated in RCTs [43] and reached similar conclusions regarding categories of interventions, with more interventions targeting parents than health care workers. In the future, further research may aim at listing all possible interventions in details.

## CONCLUSIONS

We identified numerous and heterogenous definitions, models of care and categories of intervention that have been conceptualised over time and across various settings, reflecting the broad and evolving nature of N&FCC.

Newborn and family-centred care integrates person- and family-centred care, recognising the newborn as an individual with rights, communicative capacities, and developmental needs, while also emphasising the family’s essential role in care. It aims to reduce harmful stimuli and promote well-being of both infants and families.

Our findings highlight the need for greater conceptual clarity and standardisation in this area. The synthesis provided in this review may serve as a foundation for an international working group, comprising researchers, policymakers, health professionals, and family representatives, to harmonise definitions and identify core components of care.

This review also provides practical resources for implementers by outlining existing models and intervention categories that could be adapted to diverse contexts and used to inspire new policies and practices that advance N&FCC.

Importantly, research from LMICs remain underrepresented; future studies should prioritise inclusion of LMIC stakeholders wherever possible. Additionally, we identified a significant gap in interventions aimed at supporting health care professionals, which warrants focused attention in future research and policy development.

## Additional material


Online Supplementary Document
