# Supplementary material for: Person- and family-centred care in neonatology: a scoping review to identify existing definitions, models of care, and related categories of interventions
Source: J Glob Health. 2025 Sep 26;15:04263. doi: 10.7189/jogh.15.04263 (PMC12464611; doi:10.7189/jogh.15.04263)
Supplement: Online Supplementary Document [file jogh-15-04263-s001.pdf]

Supplement to: Togo A, Lincetto O, Bua J, Mariani I, Lazzerini M. Person- and family-centred care in neonatology: a scoping review to identify existing definitions, models of care, and related categories of interventions. J Glob Health. 2025;15:04263.

Supplemental Table 1: Preferred Reporting Items for Systematic reviews and Meta-Analyses extension for Scoping Reviews (PRISMA-ScR) Checklist

SECTION	ITEM	PRISMA-ScR CHECKLIST ITEM	REPORTED ON PAGE #
**TITLE**			
Title	1	Identify the report as a scoping review.	1
**ABSTRACT**			
Structured summary	2	Provide a structured summary that includes (as applicable): background, objectives, eligibility criteria, sources of evidence, charting methods, results, and conclusions that relate to the review questions and objectives.	2
**INTRODUCTION**			
Rationale	3	Describe the rationale for the review in the context of what is already known. Explain why the review questions/objectives lend themselves to a scoping review approach.	4
Objectives	4	Provide an explicit statement of the questions and objectives being addressed with reference to their key elements (e.g., population or participants, concepts, and context) or other relevant key elements used to conceptualize the review questions and/or objectives.	6
**METHODS**			
Protocol and registration	5	Indicate whether a review protocol exists; state if and where it can be accessed (e.g., a Web address); and if available, provide registration information, including the registration number.	6
Eligibility criteria	6	Specify characteristics of the sources of evidence used as eligibility criteria (e.g., years considered, language, and publication status), and provide a rationale.	7-8
Information sources*	7	Describe all information sources in the search (e.g., databases with dates of coverage and contact with authors to identify additional sources), as well as the date the most recent search was executed.	8-9
Search	8	Present the full electronic search strategy for at least 1 database, including any limits used, such that it could be repeated.	7
Selection of sources of evidence†	9	State the process for selecting sources of evidence (i.e., screening and eligibility) included in the scoping review.	7
Data charting process‡	10	Describe the methods of charting data from the included sources of evidence (e.g., calibrated forms or forms that have been tested by the team before their use, and whether data charting was done independently or in duplicate) and any processes for obtaining and confirming data from investigators.	8-9
Data items	11	List and define all variables for which data were sought and any assumptions and simplifications made.	8-9
Critical appraisal of individual sources of evidence§	12	If done, provide a rationale for conducting a critical appraisal of included sources of evidence; describe the methods used and how this information was used in any data synthesis (if appropriate).	8-9

Synthesis of results	13	Describe the methods of handling and summarizing the data that were charted.	8-9

St. Michael's
Inspired Care.
Inspiring Science.

SECTION	ITEM	PRISMA-ScR CHECKLIST ITEM	REPORTED ON PAGE #
**RESULTS**			
Selection of sources of evidence	14	Give numbers of sources of evidence screened, assessed for eligibility, and included in the review, with reasons for exclusions at each stage, ideally using a flow diagram.	7,8, 13
Characteristics of sources of evidence	15	For each source of evidence, present characteristics for which data were charted and provide the citations.	7
Critical appraisal within sources of evidence	16	If done, present data on critical appraisal of included sources of evidence (see item 12).	9-12
Results of individual sources of evidence	17	For each included source of evidence, present the relevant data that were charted that relate to the review questions and objectives.	9-12
Synthesis of results	18	Summarize and/or present the charting results as they relate to the review questions and objectives.	9-12
**DISCUSSION**			
Summary of evidence	19	Summarize the main results (including an overview of concepts, themes, and types of evidence available), link to the review questions and objectives, and consider the relevance to key groups.	24-26
Limitations	20	Discuss the limitations of the scoping review process.	26
Conclusions	21	Provide a general interpretation of the results with respect to the review questions and objectives, as well as potential implications and/or next steps.	27
**FUNDING**			
Funding	22	Describe sources of funding for the included sources of evidence, as well as sources of funding for the scoping review. Describe the role of the funders of the scoping review.	28

JBI = Joanna Briggs Institute; PRISMA-ScR = Preferred Reporting Items for Systematic reviews and Meta-Analyses extension for Scoping Reviews.

* Where *sources of evidence* (see second footnote) are compiled from, such as bibliographic databases, social media platforms, and Web sites.

† A more inclusive/heterogeneous term used to account for the different types of evidence or data sources (e.g., quantitative and/or qualitative research, expert opinion, and policy documents) that may be eligible in a scoping review as opposed to only studies. This is not to be confused with *information sources* (see first footnote).

‡ The frameworks by Arksey and O'Malley (6) and Levac and colleagues (7) and the JBI guidance (4, 5) refer to the process of data extraction in a scoping review as data charting.

§ The process of systematically examining research evidence to assess its validity, results, and relevance before using it to inform a decision. This term is used for items 12 and 19 instead of "risk of bias" (which is more applicable to systematic reviews of interventions) to include and acknowledge the various sources of evidence that may be used in a scoping review (e.g., quantitative and/or qualitative research, expert opinion, and policy document).

Appendix 1: Search Strategy

PubMed/Medline/Web of Science

#1 TITLE/ABSTRACT
"Person-centered-care" OR "Person-centred-care" OR "family-centered-care" OR "family-centred-care" OR "IFCDC" OR "FCNDC" OR "family-integrated-care" OR "FIC" OR "family-participatory-care" OR "kangaroo-mother-care" OR "kangaroo-care" OR "patient-centered-care" OR "patient-centred-care" OR "KMC" OR "skin-to-skin" OR "mother-nicu-care" OR "rooming-in" OR "Mother-Neonatal-Intensive-Care" OR "Open-neonatal-unit" OR "Open-neonatal-care" OR "Open-neonatal-ward*" OR "Mother-NICU" OR "Nurturing-care" OR "People-centered-care" OR "People-centred-care" OR "NIDCAP" OR "FICARE" OR "developmental-care"
#2 TITLE/ABSTRACT
"Newborn*" OR "neonat*" OR "preterm" OR "pre-term" OR "prematur*" OR "infan*" OR "baby" OR "babies"
#3 TITLE/ABSTRACT
"principle*" OR "Concept*" OR "definition*" OR "guideline*" OR "standard*" OR "categor*" OR "implementation" OR "program*" OR "protocol*" OR "consensus" OR "clinical-practice*"

#4 ALL FIELDS
NOTNLM OR publisher[sb] OR inprocess[sb] OR pubmednotmedline[sb] OR indatareview[sb] OR pubstatusaheadofprint
#5 #1 AND #2 AND #3 AND #4

Google Scholar
Person centered care | Person centred care | family centered care | family centred care | IFCDC | IFCNDC | family integrated care | FIC | family participatory care | kangaroo mother care | kangaroo care | patient centered care | patient centred care | KMC | skin to skin | mother nicu care | rooming in | Mother Neonatal Intensive Care | Open neonatal unit | Open-neonatal care | Open neonatal ward* | Mother NICU | Nurturing care | People centered care | People centred care | NIDCAP | FICARE | developmental care
Newborn* | neonat* | preterm | pre-term | prematur* | infan* | baby | babies
principle* | Concept* | definition* | guideline* | standard* | categor* | implementation | program* | protocol* | consensus | clinical practice*

EMBASE

("Person centered care" OR "Person-centered care" OR "Person centred care" OR "Person-centred care" OR "family centered care" OR "family-centered care" OR "family centred care" OR "family-centred care" OR "FCC" OR "family-centered developmental care" OR "family centered developmental care" OR "Family-centered newborn developmental care" OR "Family centrered newborn developmental care" OR "Infant family-centered developmental care" OR "infant family centered developmental care" OR "IFCDC" OR "FCNDC" OR "family integrated care" OR "FIC" OR "family participatory care" OR "kangaroo mother care" OR "KMC" OR "skin-to-skin" OR "mother-nicu care" OR "mother nicu care" OR "rooming-in" OR "Mother Neonatal Intensive Care Unit" OR "Open neonatal unit" OR "Mother NICU" OR "Mother-NICU" OR "Nurturing care" OR "People centered care" OR "People centred care" OR "NIDCAP" OR "FICARE" OR "developmental care") AND (Newborn* OR "neonatal care" OR "small and sick newborns" OR "preterm") AND ("principle*" OR Concept* OR "definition*" OR guideline* OR standard* OR categor* OR "implementation") AND ([embryo]/lim OR [fetus]/lim OR [infant]/lim OR [newborn]/lim)

Appendix 2: Studies included in this review: N=91

1. Erdei C, Inder TE, Dodrill P, Woodward LJ. The Growth and Development Unit. A proposed approach for enhancing infant neurodevelopment and family-centered care in the Neonatal Intensive Care Unit. J Perinatol. 2019;39(12):1684-7.
2. Lisanti AJ, Vittner D, Medoff-Cooper B, Fogel J, Wernovsky G, Butler S. Individualized Family-Centered Developmental Care: An Essential Model to Address the Unique Needs of Infants With Congenital Heart Disease. J Cardiovasc Nurs. 2019;34(1):85-93.
3. Beebe B, Myers MM, Lee SH, Lange A, Ewing J, Rubinchik N, et al. Family nurture intervention for preterm infants facilitates positive mother-infant face-to-face engagement at 4 months. Dev Psychol. 2018;54(11):2016-31.
4. Hall SL, Hynan MT, Phillips R, Lassen S, Craig JW, Goyer E, et al. The neonatal intensive parenting unit: an introduction. J Perinatol. 2017;37(12):1259-64.
5. Ahlqvist-Bjorkroth S, Boukydis Z, Axelin AM, Lehtonen L. Close Collaboration with Parents intervention to improve parents' psychological well-being and child development: Description of the intervention and study protocol. Behav Brain Res. 2017;325(Pt B):303-10.
6. Chan GJ, Valsangkar B, Kajeepeta S, Boundy EO, Wall S. What is kangaroo mother care? Systematic review of the literature. J Glob Health. 2016;6(1):010701.
7. Welch MG, Firestein MR, Austin J, Hane AA, Stark RI, Hofer MA, et al. Family Nurture Intervention in the Neonatal Intensive Care Unit improves social-relatedness, attention, and neurodevelopment of preterm infants at 18 months in a randomized controlled trial. J Child Psychol Psychiatry. 2015;56(11):1202-11.
8. Welch MG, Hofer MA, Stark RI, Andrews HF, Austin J, Glickstein SB, et al. Randomized controlled trial of Family Nurture Intervention in the NICU: assessments of length of stay, feasibility and safety. BMC Pediatr. 2013;13:148.
9. Staniszewska S, Brett J, Redshaw M, Hamilton K, Newburn M, Jones N, et al. The POPPY study: developing a model of family-centred care for neonatal units. Worldviews Evid Based Nurs. 2012;9(4):243-55.
10. Welch MG, Hofer MA, Brunelli SA, Stark RI, Andrews HF, Austin J, et al. Family nurture intervention (FNI): methods and treatment protocol of a randomized controlled trial in the NICU. BMC Pediatr. 2012;12:14.
11. Als H, McAnulty GB. The Newborn Individualized Developmental Care and Assessment Program (NIDCAP) with Kangaroo Mother Care (KMC): Comprehensive Care for Preterm Infants. Curr Womens Health Rev. 2011;7(3):288-301.
12. Mikkelsen G, Frederiksen K. Family-centred care of children in hospital - a concept analysis. J Adv Nurs. 2011;67(5):1152-62.
13. Levin A. The Mother-Infant unit at Tallinn Children's Hospital, Estonia: a truly baby-friendly unit. Birth. 1994;21(1):39-44, discussion 5-6.
14. Franck LS, Hodgson C, Gay CL, Bisgaard R, Cormier DM, Joe P, et al. Mobile-Enhanced Family-Integrated Care for Preterm Infants: Nurse and Physician Views About Implementation.
15. Pricoco R, Mayer-Huber S, Paulick J, Benstetter F, Zeller M, Keller M. Impact of a family-centred clinical care programme on short-term outcomes of very low-birth weight infants. Acta Paediatr. 2023;112(11):2368-77.
16. Lisanti AJ, Vittner DJ, Peterson J, Van Bergen AH, Miller TA, Gordon EE, et al. Developmental care pathway for hospitalised infants with CHD: on behalf of the Cardiac

Newborn Neuroprotective Network, a Special Interest Group of the Cardiac Neurodevelopmental Outcome Collaborative. Cardiol Young. 2023;33(12):2521-38.
17. Dien R, Benzies KM, Zanoni P, Kurilova J. Alberta Family Integrated Care TM and Standard Care: A Qualitative Study of Mothers' Experiences of their Journeying to Home from the Neonatal Intensive Care Unit.
18. Aita M, Heon M, Lavallee A, De Clifford Faugere G, Altit G, Le May S, et al. Nurturing and quiet intervention (NeuroN-QI) on preterm infants' neurodevelopment and maternal stress and anxiety: A pilot randomized clinical trial protocol. J Adv Nurs. 2021;77(7):3192-203.
19. Franck LS, Waddington C, O'Brien K. Family Integrated Care for Preterm Infants. Crit Care Nurs Clin North Am. 2020;32(2):149-65.
20. de Salaberry J, Hait V, Thornton K, Bolton M, Abrams M, Shivananda S, et al. Journey to mother baby care: Implementation of a combined care/couplet model in a Level 2 neonatal intensive care unit. Birth Defects Res. 2019;111(15):1060-72.
21. Peterson JK, Evangelista LS. Developmentally Supportive Care in Congenital Heart Disease: A Concept Analysis. J Pediatr Nurs. 2017;36:241-7.
22. Macho P. Individualized Developmental Care in the NICU: A Concept Analysis. Adv Neonatal Care. 2017;17(3):162-74.
23. Lebel V, Aita M. Analyse du concept « soins du développement » selon la méthode basée sur les principes. Recherche en soins infirmiers. 2013;N° 113(2):34-42.
24. WHO Who. Standards for improving quality of care for small and sick newborns in health facilities. Geneva2020.
25. Committee On Hospital C, Institute For P, Family-Centered C. Patient- and family-centered care and the pediatrician's role. Pediatrics. 2012;129(2):394-404.
26. O'Brien K, Bracht M, Macdonell K, McBride T, Robson K, O'Leary L, et al. A pilot cohort analytic study of Family Integrated Care in a Canadian neonatal intensive care unit. BMC Pregnancy Childbirth. 2013;13 Suppl 1(Suppl 1):S12.
27. O'Brien K, Robson K, Bracht M, Cruz M, Lui K, Alvaro R, et al. Effectiveness of Family Integrated Care in neonatal intensive care units on infant and parent outcomes: a multicentre, multinational, cluster-randomised controlled trial. Lancet Child Adolesc Health. 2018;2(4):245-54.
28. Waddington C, van Veenendaal NR, O'Brien K, Patel N, International Steering Committee for Family Integrated C. Family integrated care: Supporting parents as primary caregivers in the neonatal intensive care unit. Pediatr Investig. 2021;5(2):148-54.
29. Ansari NS, Franck LS, Tomlinson C, Colucci A, O'Brien K. A Pilot Study of Family-Integrated Care (FICare) in Critically Ill Preterm and Term Infants in the NICU: FICare Plus. Children (Basel). 2023;10(8).
30. McAnulty G, Duffy FH, Butler S, Parad R, Ringer S, Zurakowski D, et al. Individualized developmental care for a large sample of very preterm infants: health, neurobehaviour and neurophysiology. Acta Paediatr. 2009;98(12):1920-6.
31. Als H, Duffy FH, McAnulty G, Butler SC, Lightbody L, Kosta S, et al. NIDCAP improves brain function and structure in preterm infants with severe intrauterine growth restriction. J Perinatol. 2012;32(10):797-803.
32. Coughlin ME. Age-Appropriate Care of the Premature and Hospitalized Infant. In: Kenner C, Lott JW, editors. New York: Springer Publishing Company. p. 943-6.

33. Gibbins S, Hoath SB, Coughlin M, Gibbins A, Franck L. The universe of developmental care: a new conceptual model for application in the neonatal intensive care unit. Adv Neonatal Care. 2008;8(3):141-7.
34. Larocque C, Peterson WE, Squires JE, Mason-Ward M, Mayhew K, Harrison D. Family-centred care in the Neonatal Intensive Care Unit: A concept analysis and literature review. Journal of Neonatal Nursing. 2021;27(6):402-11.
35. Ramezani T, Hadian Shirazi Z, Sabet Sarvestani R, Moattari M. Family-centered care in neonatal intensive care unit: a concept analysis. Int J Community Based Nurs Midwifery. 2014;2(4):268-78.
36. Hutchfield K. Family-centred care: a concept analysis. J Adv Nurs. 1999;29(5):1178-87.
37. Davidson JE, Aslakson RA, Long AC, Puntillo KA, Kross EK, Hart J, et al. Guidelines for Family-Centered Care in the Neonatal, Pediatric, and Adult ICU. Critical Care Medicine. 2017;45(1):103-28.
38. Shelton T. Family-centered care for children with special health care needs: ERIC; 1987.
39. White-Traut R, Norr K. An ecological model for premature infant feeding. J Obstet Gynecol Neonatal Nurs. 2009;38(4):478-89; quiz 89-90.
40. EFCNI Efftconi. European standards of care for Newborn Health: Infant-& family-centered development care 2018.
41. Melnyk BM, Feinstein NF, Alpert-Gillis L, Fairbanks E, Crean HF, Sinkin RA, et al. Reducing premature infants' length of stay and improving parents' mental health outcomes with the Creating Opportunities for Parent Empowerment (COPE) neonatal intensive care unit program: a randomized, controlled trial. Pediatrics. 2006;118(5):e1414-27.
42. Asai H. Family-Centered Care in Perinatal and Pediatric Healthcare:
A Concept Analysis. Japan journal of nursing science : JJNS. 2013;33(4):13–23.
43. Gooding JS, Cooper LG, Blaine AI, Franck LS, Howse JL, Berns SD. Family support and family-centered care in the neonatal intensive care unit: origins, advances, impact. Semin Perinatol. 2011;35(1):20-8.
44. Patel N, Ballantyne A, Bowker G, Weightman J, Weightman S. Family Integrated Care: changing the culture in the neonatal unit. Arch Dis Child. 2018;103(5):415-9.
45. Banerjee J, Aloysius A, Mitchell K, Silva I, Rallis D, Godambe SV, et al. Improving infant outcomes through implementation of a family integrated care bundle including a parent supporting mobile application. Arch Dis Child Fetal Neonatal Ed. 2020;105(2):172-7.
46. Shields L, Pratt J, Hunter J. Family centred care: a review of qualitative studies. J Clin Nurs. 2006;15(10):1317-23.
47. Melnyk BM, Feinstein NF. Reducing hospital expenditures with the COPE (Creating Opportunities for Parent Empowerment) program for parents and premature infants: an analysis of direct healthcare neonatal intensive care unit costs and savings. Nurs Adm Q. 2009;33(1):32-7.
48. Puthussery S, Chutiyami M, Tseng PC, Kilby L, Kapadia J. Effectiveness of early intervention programs for parents of preterm infants: a meta-review of systematic reviews. BMC Pediatr. 2018;18(1):223.
49. Phillips C. Family-centered maternity care: books.google.com; 2003.
50. Aita M, Snider L. The art of developmental care in the NICU: a concept analysis. J Adv Nurs. 2003;41(3):223-32.

51. Britto P, Lye S, Proulx K, Yousafzai A, Matthews S, ... Nurturing care: promoting early childhood development. The Lancet. 2017.
52. Brown W, Pearl LF, Carrasco N. Evolving models of family-centered services in neonatal intensive care. Children's health care : journal of the Association for the Care of Children's Health. 1991;20(1):50-5.
53. Charpak N, Ruiz JG. Kmc, concepts, definitions and praxis: What elements are applicable in what settings in which local circumstances? Current Women's Health Reviews. 2011;7(3):232-42.
54. Czynski AJ, Souza M, Lechner BE. The Mother Baby Comfort Care Pathway: The Development of a Rooming-In-Based Perinatal Palliative Care Program. Advances in neonatal care : official journal of the National Association of Neonatal Nurses. 2022;22(2):119-24.
55. Franck LS, O'Brien K. The evolution of family-centered care: From supporting parent-delivered interventions to a model of family integrated care. Birth Defects Res. 2019;111(15):1044-59.
56. Klemming S, Lilliesköld S, Arwehed S, Jonas W, Lehtonen L, Westrup B. Mother-newborn couplet care: Nordic country experiences of organization, models and practice. J Perinatol. 2023;43(Suppl 1):17-25.
57. Pineda R, Kellner P, Ibrahim C, Smith J. Supporting and Enhancing NICU Sensory Experiences (SENSE), 2nd Edition: An Update on Developmentally Appropriate Interventions for Preterm Infants. Children. 2023;10(6) (no pagination).
58. McAlinden B, Pool N, Harnischfeger J, Waak M, Campbell M. 'Baby Liberation' - Developing and implementing an individualised, developmentally-supportive care bundle to critically unwell infants in an Australian Paediatric Intensive Care Unit. Early Hum Dev. 2024;190:105944.
59. Nyqvist KH, Anderson GC, Bergman N, Cattaneo A, Charpak N, Davanzo R, et al. State of the art and recommendationsKangaroo mother care: Application in a high-tech environment. Acta Paediatrica, International Journal of Paediatrics. 2010;99(6):812-9.
60. Soni R, Tscherning C. Family-centred and developmental care on the neonatal unit. Paediatrics and Child Health (United Kingdom). 2021;31(1):18-23.
61. Westrup B. Family-centered developmentally supportive care: The Swedish example. Archives de Pediatrie. 2015;22(10):1086-91.
62. Murphy M, Shah V, Benzies K. Effectiveness of Alberta Family-Integrated Care on Neonatal Outcomes: A Cluster Randomized Controlled Trial. J Clin Med. 2021;10(24).
63. Als H, Lawhon G, Duffy FH, McAnulty GB, Gibes-Grossman R, Blickman JG. Individualized developmental care for the very low-birth-weight preterm infant. Medical and neurofunctional effects. Jama. 1994;272(11):853-8.
64. Als H. Developmental care in the newborn intensive care unit. Curr Opin Pediatr. 1998;10(2):138-42.
65. Chellani H, Arya S, Mittal P, Bahl R. Mother-Newborn Care Unit (MNCU) Experience in India: A Paradigm Shift in Care of Small and Sick Newborns. Indian J Pediatr. 2022;89(5):484-9.
66. Chellani H, Mittal P, Arya S. Mother-Neonatal Intensive Care Unit (M-NICU): A Novel Concept in Newborn Care. Indian Pediatr. 2018;55(12):1035-6.
67. Liu WF, Laudert S, Perkins B, Macmillan-York E, Martin S, Graven S, et al. The development of potentially better practices to support the neurodevelopment of infants in the NICU. J Perinatol. 2007;27 Suppl 2:S48-74.

68. Shuman CA-O, Morgan M Fau - Vance A, Vance A. Integrating Neonatal Intensive Care Into a Family Birth Center: Describing the Integrated NICU (I-NIC). LID - 10.1097/JPN.0000000000000759 [doi]. J Perinat Neonatal Nurs
. 2023(1550-5073 (Electronic)).
69. Kapito EM, Chirwa EM, Chodzaza E, Norr KF, Patil C, Maluwa AO, et al. The H-HOPE behavioral intervention plus Kangaroo Mother Care increases mother-preterm infant responsivity in Malawi: a prospective cohort comparison. BMC Pediatr. 2023;23(1):187.
70. Schuetz Haemmerli N, von Gunten G, Khan J, Stoffel L, Humpl T, Cignacco E. Interprofessional Collaboration in a New Model of Transitional Care for Families with Preterm Infants - The Health Care Professional's Perspective. J Multidiscip Healthc. 2021;14:897-908.
71. Mhango P, Chipeta E, Muula AS, Robb-McCord J, White P, Litch JA, et al. Implementing the Family-Led Care model for preterm and low birth weight newborns in Malawi: Experience of healthcare workers. Afr J Prim Health Care Fam Med. 2020;12(1):e1-e11.
72. Altimier L, Kenner C, Damus K. The Wee Care Neuroprotective NICU Program (Wee Care): The Effect of a Comprehensive Developmental Care Training Program on Seven Neuroprotective Core Measures for Family-Centered Developmental Care of Premature Neonates. Newborn and Infant Nursing Reviews. 2015;15(1):6-16.
73. Altimier L, Phillips R. Neuroprotective Care of Extremely Preterm Infants in the First 72 Hours After Birth. Crit Care Nurs Clin North Am. 2018;30(4):563-83.
74. Altimier L, Phillips R. The Neonatal Integrative Developmental Care Model: Advanced Clinical Applications of the Seven Core Measures for Neuroprotective Family-centered Developmental Care. Newborn and Infant Nursing Reviews. 2016;16(4):230-44.
75. WHO WHO. Global position paper. Kangaroo mother care: a transformative innovation in health care. Geneva. 2023.
76. Care IfPaFC. What is PFCC: IPFCC; 2024 [Available from: https://www.ipfcc.org/about/pfcc.html.
77. LaRonde MP, Connor JA, Cerrato B, Chiloyan A, Lisanti AJ. Individualized Family-Centered Developmental Care for Infants With Congenital Heart Disease in the Intensive Care Unit. Am J Crit Care. 2022;31(1):e10-e9.
78. Torowicz D, Lisanti AJ, Rim JS, Medoff-Cooper B. A developmental care framework for a cardiac intensive care unit: a paradigm shift. Advances in neonatal care : official journal of the National Association of Neonatal Nurses. 2012;12 Suppl 5:S28-32.
79. Browne JV, Jaeger CB, Kenner C, Gravens Consensus Committee on I, Family Centered Developmental C. Executive summary: standards, competencies, and recommended best practices for infant- and family-centered developmental care in the intensive care unit. J Perinatol. 2020;40(Suppl 1):5-10.
80. Browne JV, White RD. Foundations of developmental care. Clin Perinatol. 2011;38(4):xv-xvii.
81. Griffiths N, Spence K, Loughran-Fowlds A, Westrup B. Individualised developmental care for babies and parents in the NICU: Evidence-based best practice guideline recommendations. Early Hum Dev. 2019;139:104840.
82. Coughlin M, Gibbins S, Hoath S. Core measures for developmentally supportive care in neonatal intensive care units: theory, precedence and practice. J Adv Nurs. 2009;65(10):2239-48.

83. Black MM. Nurturing Care Framework and Implementation Science: Promoting Nutrition, Health and Development among Infants and Toddlers Globally. Nestle Nutrition Institute workshop series. 2019;92:53-64.
84. Black MM, Trude ACB, Lutter CK. All Children Thrive: Integration of Nutrition and Early Childhood Development. Annu Rev Nutr. 2020;40:375-406.
85. Black MM, Behrman JR, Daelmans B, Prado EL, Richter L, Tomlinson M, et al. The principles of Nurturing Care promote human capital and mitigate adversities from preconception through adolescence. BMJ Glob Health. 2021;6(4).
86. Klemming S, Lillieskold S, Westrup B. Mother-Newborn Couplet Care from theory to practice to ensure zero separation for all newborns. Acta Paediatr. 2021;110(11):2951-7.
87. Craig JW, Glick C, Phillips R, Hall SL, Smith J, Browne J. Recommendations for involving the family in developmental care of the NICU baby. (1476-5543 (Electronic)).
88. Landsem IP, Handegård BH, Tunby J, Ulvund SE, Rønning JA. Early intervention program reduces stress in parents of preterms during childhood, a randomized controlled trial. Trials. 2014;15:387.
89. Newnham CA, Milgrom J, Skouteris H. Effectiveness of a modified Mother-Infant Transaction Program on outcomes for preterm infants from 3 to 24 months of age. Infant Behav Dev. 2009;32(1):17-26.
90. Maria A, Agrawal D. Family-Centered Care for Newborns: From Pilot Implementation to National Scale-up in India. Indian Pediatr. 2021;58 Suppl 1:S60-s3.
91. Maria A, Upadhyay S, Vallomkonda N. Nurturing Beyond the Womb - Early Intervention Practices in Newborn Care Unit. Indian Pediatr. 2021;58 Suppl 1:S53-s9.

Supplemental Table 2: List of Definitions

	Name of model of care	Definition found in paper<br><br>(reference)
1	**I-NIC**<br><br>**Integrating Neonatal Intensive Care Into a Family Birth Center**	FCC is predicated on the following principles: (a) parents should not be separated from their hospitalized infant; (b) parents and healthcare providers collaborate in caring for the child; and (c) all caregiving should facilitate parental involvement.<br>Another recent and novel development that combines environmental redesign and FCC practices, aiming to mitigate the stress of maternal-infant separation, is the integrated neonatal intensive care (I-NIC) model.<br>I-NIC began with a redesign of the traditional NICU care space, similar to the redesign of labor and delivery units into all-inclusive care rooms (eg, labor, delivery, recovery, and postpartum [LDRP]). When redesigning the NICU environment for the I-NIC model, FCC principles guided the creation of a unit or rooms where LDRP and NIC are provided together in the same room.<br>I-NIC incorporates the qualities of LDRP, FCC, and SFRs to create an environment where infants requiring intensive care remain with their mothers in the same LDRP room for the duration of their hospitalization. Thus, the I-NIC model mitigates maternal-infant separation for infants requiring intensive care. In the I-NIC model of care, the mother may be discharged from medical care after recovery from delivery but may continue to stay with her infant until discharge. Regardless of which patient, mother or infant, is ready to be discharged first, both remain together in the same room for the duration of their hospitalization.<br><br>(Shuman CJ, Morgan M, Vance A. Integrating Neonatal Intensive Care Into a Family Birth Center: Describing the Integrated NICU (I-NIC). J Perinat Neonatal Nurs. 2023 Sep 28. doi: 10.1097/JPN.0000000000000759. Epub ahead of print. PMID: 37773333.)
2	**H-HOPE**<br><br>**Hospital to Home: Optimizing the Preterm Infant Environment**	The only early behavioral intervention for preterm infants with both well-established efficacy and a standardized protocol is H-HOPE (Hospital to Home: Optimizing the Preterm Infant Environment). H-HOPE has both parent- and infant-focused components. The infant-focused component was developed first and is a multisensory intervention that provides auditory, tactile, visual, and vestibular stimulation. Originally called ATVV, the infant component is now called Massage + for easier recognition by parents. Because parents of preterm infants continued to experience high stress and anxiety, H-HOPE added a component for parents called Parents + , which uses participatory guidance to help parents to read, interpret and respond to preterm infant cues and provide Massage + for their infant.<br><br>(Kapito EM, Chirwa EM, Chodzaza E, Norr KF, Patil C, Maluwa AO, White-Traut R. The H-HOPE behavioral intervention plus Kangaroo Mother Care increases mother-preterm infant responsivity in Malawi: a prospective cohort comparison. BMC Pediatr. 2023 Apr 21;23(1):187. doi: 10.1186/s12887-023-04015-z. PMID: 37085764; PMCID: PMC10120231.)
3	**Mother–newborn care unit**	Mother–newborn care unit (MNCU) or mother–newborn intensive care unit (M–NICU) is a facility where sick and small newborns are cared with their mothers 24/7 with all facilities of level II newborn care and provision for postnatal care to mothers. The mother is not a mere visitor, but she has her bed inside the NICU. Mother as a resident of MNCU becomes an active caregiver and is involved in continuum of neonatal care. Mothers in MNCU substantially contribute to care of babies including feeding, changing diapers, and monitoring babies for danger signs. MNCU provides opportunity for mother to be the primary caregiver in MNCU, thus providing family-centered developmentally supportive care to newborns. Presence of mother in MNCU gives ample opportunity to health care personnel to teach the mothers, healthy practices of neonatal care thus preparing them for taking care of neonates after discharge. Last but not the least, MNCU resulted in mother–newborn couplet care by pediatrician and obstetrician with better co-ordination of neonatal and maternal care.<br><br>(Chellani H, Arya S, Mittal P, Bahl R. Mother-Newborn Care Unit (MNCU) Experience in India: A Paradigm Shift in Care of Small and Sick Newborns. Indian J Pediatr. 2022 May;89(5):484-489. doi: 10.1007/s12098-022-04145-9. Epub 2022 Mar 4. PMID: 35244878; PMCID: PMC8895087.
4	**MotherBaby Care**	MotherBaby Care in a Level 2 neonatal intensive care unit (NICU) is an adaptation of the postpartum couplet model where the mother infant dyad is considered inseparable

		and where mothers are supported to be the primary caregivers for their baby. This family centered model of care builds on the experiences from the Mother-Infant Unit in Estonia and Sweden, and the family integrated model of care in Toronto. MotherBaby Care supports breastfeeding and aligns with the Baby-Friendly Hospital Initiative 10 Steps to Successful Breastfeeding. A key feature of MotherBaby Care is the coordination of care since one nurse provides care to both mother and infant. In MotherBaby Care the nurse is well positioned to optimize the coordination of care that supports the mother and infant's recovery and promote the development of the maternal/infant relationship. MotherBaby Care eliminates separations, reduces transitions in care, and improves the continuity of care; thereby providing an effective and efficient quality care experience that can reduce length stay and improve communication. In MotherBaby Care, the mother is discharged from medical care according to her care needs, and continues to stay with her baby until discharged home regardless of which patient, mother or baby, is ready first.<br><br>(de Salaberry J, Hait V, Thornton K, Bolton M, Abrams M, Shivananda S, Kiarash M, Osiovich H. Journey to mother baby care: Implementation of a combined care/couplet model in a Level 2 neonatal intensive care unit. Birth Defects Res. 2019 Sep 1;111(15):1060-1072. doi: 10.1002/bdr2.1524. Epub 2019 May 27. PMID: 31132224.
5	**Newborn Intensive Parenting Unit (NIPU)**	The term Newborn Intensive Parenting Unit (NIPU) was derived to capture this evolution in goals. This model, which began in the 'We Are Family' Homeroom of the Vermont Oxford Network (VON) and has been the topic of presentations at the VON Annual Quality Congress, is based on evolving concepts of family centered and family-integrated care (FICare). One component of the NIPU is FICare, a model of care where parents are intimately involved in their sick or premature baby's care for as many hours a day as possible. While FICare serves as the foundation of the NIPU, a set of potentially better practices (PBPs) represent the building blocks surrounding and supporting both parents and staff. We describe six areas of PBPs, along with their empirical foundations, which are derived from the 'Interdisciplinary Recommendations for the Psychosocial Support of NICU Parents'8 published by the National Perinatal Association in collaboration with many other professional and parent organizations. These include: (1) family centered developmental care, (2) peer support, (3) mental health support, (4) palliative and bereavement care, (5) post-discharge support and (6) staff education and support.<br><br>(Hall SL, Hynan MT, Phillips R, Lassen S, Craig JW, Goyer E, Hatfield RF, Cohen H. The neonatal intensive parenting unit: an introduction. J Perinatol. 2017 Dec;37(12):1259-1264. doi: 10.1038/jp.2017.108. Epub 2017 Aug 10. PMID: 28796241; PMCID: PMC5718987.)
6	**Family Nurture Intervention (FNI)**	Family Nurture Intervention (FNI) is a new intervention designed to overcome the maladaptive conditioning effects of maternal separation and the NICU environment on the premature infant. It is hypothesized to do so by facilitating an emotional connection and by establishing an adaptive classical homeostatic conditioning routine between mother and infant, referred to as the Calming Cycle.<br><br>(Welch MG, Firestein MR, Austin J, Hane AA, Stark RI, Hofer MA, Garland M, Glickstein SB, Brunelli SA, Ludwig RJ, Myers MM. Family Nurture Intervention in the Neonatal Intensive Care Unit improves social-relatedness, attention, and neurodevelopment of preterm infants at 18 months in a randomized controlled trial. J Child Psychol Psychiatry. 2015 Nov;56(11):1202-11. doi: 10.1111/jcpp.12405. Epub 2015 Mar 11. PMID: 25763525
7	**Transition to Home**	The "Transition to Home (TtH)"-model offers structured, individual support for families with preterm infants before and after hospital discharge. It was developed to support families with preterm infants, beginning at birth and ending six months after hospital discharge. The model adheres to principles of integrated care, including interventions and processes to improve interprofessional collaboration between stakeholders in the health care system and ensure that treatment and care of patients is coordinated optimally along the treatment continuum. The "Transition to Home (TtH)" model is designed to optimize transitional care of families with preterm infants between 24 and 35 weeks of gestational age by giving parents and infants structured, individual support, improving parental mental health and competence, promoting the child's development, and optimizing interprofessional collaboration (IPC).<br><br>(Schuetz Haemmerli N, von Gunten G, Khan J, Stoffel L, Humpl T, Cignacco E. Interprofessional Collaboration in a New Model of Transitional Care for Families with Preterm Infants - The Health Care Professional's Perspective. J Multidiscip Healthc. 2021 Apr 23;14:897-908. doi: 10.2147/JMDH.S303988. PMID: 33935499; PMCID: PMC8079346.)

8	**Family-Led Care model**	The Family-Led Care model positions families as active, confident participants in the care of their preterm and LBW babies in the health facility and at home. Family members are the central role-players that constitute the continuum between community and healthcare facility and lead the health-seeking behaviour in antenatal and postnatal care. The model promotes improved quality of care at the facility level and increased access to and utilisation of care through a functional referral system to address morbidity and mortality of preterm and LBW babies after discharge from the health facility.<br><br>(Mhango P, Chipeta E, Muula AS, Robb-McCord J, White P, Litch JA, Kamanga I, Freeman R, Bergh AM. Implementing the Family-Led Care model for preterm and low birth weight newborns in Malawi: Experience of healthcare workers. Afr J Prim Health Care Fam Med. 2020 Aug 17;12(1):e1-e11. doi: 10.4102/phcfm.v12i1.2266).
9	**Growth and Development Unit**	Our team has developed a specialized, neurodevelopmentally-focused program embedded within a 66-bed level III NICU, known as the Growth and Development Unit (GDU). The GDU was conceptualized by a transdisciplinary group of clinicians and parents, with a mission to provide comprehensive, individualized, developmentally supportive health care services for infants and families who transition beyond the acute critical illness phase. A full spectrum of services and neurodevelopmental support are offered to GDU infants and families, including highly specialized medical and nursing care, feeding therapy, nutrition guidance, lactation counseling, developmental therapy, care coordination, and family mental health and wellness support, in keeping with the key principles of family-centered developmental care for ICU settings.<br><br>(Erdei C, Inder TE, Dodrill P, Woodward LJ. The Growth and Development Unit. A proposed approach for enhancing infant neurodevelopment and family-centered care in the Neonatal Intensive Care Unit. J Perinatol. 2019 Dec;39(12):1684-1687. doi: 10.1038/s41372-019-0514-7. Epub 2019 Oct 3. PMID: 31582813.)
10	**POPPY project (Parents of Premature Babies Project)**	The POPPY project (Parents of Premature Babies Project) emerged from a group of parents who felt that, while their babies had received good quality clinical care, they themselves had poor experiences of the neonatal unit. In many respects they felt they had received "non-family–centred care," resonating with studies that have identified difficulties with properly implementing family-centred care in practice. The POPPY project conceptualised family-centred care as a philosophy of care, with key principles and as a model of care with core components, which can be implemented. Parents also recognised the importance of synthesising data about parental experiences with research evidence from a systematic review that identified effective interventions.<br><br>(Staniszewska S, Brett J, Redshaw M, Hamilton K, Newburn M, Jones N, Taylor L. The POPPY study: developing a model of family-centred care for neonatal units. Worldviews Evid Based Nurs. 2012 Dec;9(4):243-55. doi: 10.1111/j.1741-6787.2012.00253.x. Epub 2012 Jun 14. PMID: 22698274.)
11	**NeoPAss**	We established a multi-component FCC clinical care pathway and case management programme named NeoPAss in the department of neonatology in the children's hospital Passau. Key elements of the programme include a FCC clinical care pathway and case management. In short, case managers were appointed to be responsible for each individual family already before preterm birth as soon as a pregnancy at risk was detected. The programme was implemented by a structured change management, staff training and mandatory participation of all staff members involved in neonatal care. Additionally, infrastructural interventions were implemented to tend to parental needs such as unlimited access for parents to their newborns at the NICU and rooming-in facilities.<br><br>(Pricoco R, Mayer-Huber S, Paulick J, Benstetter F, Zeller M, Keller M. Impact of a family-centred clinical care programme on short-term outcomes of very low-birth weight infants. Acta Paediatr. 2023 Nov;112(11):2368-2377. doi: 10.1111/apa.16944. Epub 2023 Sep 1. PMID: 37551158.)
12	**Alberta FICare™**	The goal of Alberta FICare™ is change in culture and practice that involves and supports parents in their role while their infant is receiving care in a level II NICU. Alberta FICare™ is a theoretically driven, dynamic, psychoeducational model of care that empowers parents to build their knowledge, skill, and confidence in caring for their infant(s) in the NICU to prepare for earlier discharge. The model has 3 main components: Relational Communication, Parent Education, and Parent Support.

		Murphy M, Shah V, Benzies K. Effectiveness of Alberta Family-Integrated Care on Neonatal Outcomes: A Cluster Randomized Controlled Trial. J Clin Med. 2021;10(24)
13	**NeuroN-QI**	NeuroN-QI, a nurturing and quiet intervention to promote infant neurodevelopment. NeuroN-QI is an intervention (I) which consists of nurturing periods (N) between mother–infant dyads (skin-to- skin contact (SSC) and auditory stimulation) to promote maternal physical and emotional proximity, and quiet periods (Q) (controlled light and noise levels as well as olfactory stimulation in incubators/cribs) to promote infants' neurodevelopment (Neuro).<br><br>(Aita M, Héon M, Lavallée A, De Clifford Faugère G, Altit G, Le May S, Dorval V, Lippé S, Larone Juneau A, Remmer E, Rennick JE. Nurturing and quiet intervention (NeuroN-QI) on preterm infants' neurodevelopment and maternal stress and anxiety: A pilot randomized clinical trial protocol. J Adv Nurs. 2021 Jul;77(7):3192-3203. doi: 10.1111/jan.14819. Epub 2021 Mar 14. PMID: 33719093.)
14	**Neonatal Integrative Developmental Care Model**	The Neonatal Integrative Developmental Care model (IDC) combines evidence-based practices with seven neuroprotective core measures for family-centered developmentally supportive care aimed at standardizing neuroprotective care practices in the NICU. It is a framework that guides clinical practice in many neonatal intensive care units (NICUs) around the globe.<br>Neuroprotective developmentally supportive care includes creating a in the infant's care and development healing environment that manages stress and pain while offering a calming and soothing approach that keeps the whole family involved.<br><br>The Neonatal Integrative Developmental Care Model (IDC) identifies seven distinct core measures that provide clinical guidance for NICU staff in delivering neuroprotective family-centered developmental care to preterm infants and their families in the NICU. The seven neuroprotective core measures are depicted as overlapping petals of a lotus as the 1) healing environment, 2) partnering with families, 3) positioning & handling, 4) Safeguarding sleep, 5) minimizing stress and pain, 6) protecting skin, and 7) optimizing nutrition. Skin to Skin Contact (SSC) is considered the foundation for care of infants in the NICU and its importance as the "normal environment" and the ideal place of care is described. The mother/child dyad is the center of the lotus surrounded closely by symbols representing various aspects of the healing environment, highlighting the physical, extra-uterine environment in which the infant now lives, the significance of the developing infant's sensory system, and the influence of people (patient, family, and staff)who help to create a healing environment for hospitalized infants and their families. The Neonatal Integrative Developmental Care Model utilizes neuroprotective interventions as strategies to support optimal synaptic neural connections, promote normal neurological, physical, and emotional development and prevent disabilities.<br><br>(Altimier L, Phillips R Newborn and Infant The Neonatal Integrative Developmental Care Model: Advanced Clinical Applications of the Seven Core Measures for Neuroprotective Family-centered Developmental Care Nursing Reviews (2016) 16(4) 230-244.)
15	**Supporting and Enhancing NICU Sensory Experiences (SENSE) program**	The Supporting and Enhancing NICU Sensory Experiences (SENSE) program promotes consistent, age-appropriate, responsive, and evidence-based positive sensory exposures for preterm infants each day of NICU hospitalization to optimize infant and parent outcomes.<br>The SENSE program was also designed to encourage parents to be the providers of positive sensory exposures to their infants<br><br>(Pineda, R., Kellner, P., Ibrahim, C., Sense Advisory Team Working Group, & Smith, J. (2023). Supporting and Enhancing NICU Sensory Experiences (SENSE), 2nd Edition: An Update on Developmentally Appropriate Interventions for Preterm Infants. Children (Basel, Switzerland), 10(6), 961. https://doi.org/10.3390/children10060961)
16	**Baby Liberation**	Baby Liberation is an individualised IFCDC approach to providing critical care interventions within the Queensland Children's Hospital PICU. Baby Liberation aims to create a comprehensive care framework that encompasses the principles of developmental care within the broader context of PICU care.

		(McAlinden, B., et al. (2024). "'Baby Liberation' - Developing and implementing an individualised, developmentally-supportive care bundle to critically unwell infants in an Australian Paediatric Intensive Care Unit." Early Hum Dev 190: 105944.)
17	**Developmentally supportive care for infants and children with Congenital Heart Disease**	Developmentally supportive care for infants and children with CHD is family-centered nursing care that is adapted to meet the individual's unique needs as the individual interrelates with their environment. Developmentally supportive care for infants and children with CHD incorporates unique understanding of CHD and its effects on both the child and their family in order to provide excellent family-centered care through interprofessional collaboration and appropriate resources that promotes improved development, family performance, and satisfaction with care.<br><br>(Peterson, J. K. and L. S. Evangelista (2017). "Developmentally Supportive Care in Congenital Heart Disease: A Concept Analysis." J Pediatr Nurs **36**: 241-247.)
18	**Family-centered care in Perinatal and Pediatric Healthcare**	The concept of family-centered care was defined as a continuous and comprehensive care process, including information sharing about child's care, supporting decision-making and empowerment of families, based on a partnership and between families and healthcare providers, carried out with respect and dignity.<br><br>(Asai, Hiromi. "Family-Centered Care in Perinatal and Pediatric Healthcare: A Concept Analysis." *Journal of Japan Academy of Nursing Science* 33.4 (2013): 4_13-4_23.)
19	**Family-Centered Care in Neonatal Intensive Care Unit**	FCC is defined as an inter-disciplinary, comprehensive, and holistic care of neonates and families with maintaining their respect and dignity. Family, as a constant member in neonate's life and one of the main participants in healthcare, collaborates mutually with healthcare workers. Complete information exchange without any bias leads to promotion of quality of cares provided for neonates and their families.<br><br>(Ramezani T, Hadian Shirazi Z, Sabet Sarvestani R, Moattari M. Family-centered care in neonatal intensive care unit: a concept analysis. Int J Community Based Nurs Midwifery. 2014;2(4):268-78.)
20	**Individualized developmental care**	IDC is defined as an approach to providing care to infants in the NICU and their family based on their individual cues and responses to care. Care provided needs to be individualized, and collaborative between the infant, their family, and all healthcare personnel. Care also needs to include the ability to alter the infants' care and environment in an effort to decrease stress to the infant and their family and maximize neurological, cognitive, and behavioral outcomes.<br><br>(Macho P. Individualized Developmental Care in the NICU: A Concept Analysis. Adv Neonatal Care. 2017 Jun;17(3):162-174. doi: 10.1097/ANC.0000000000000374. PMID: 28030365.)
21	**Family-centred care of children in hospital**	FCC is the professional support of the child and the family through a process of involvement and participation, underpinned by empowerment and negotiation. FCC is characterized by a relationship between healthcare professionals and the family, in which both parts engage in sharing the responsibility for the child's health care.<br><br>(Mikkelsen G, Frederiksen K. Family-centred care of children in hospital - a concept analysis. J Adv Nurs. 2011 May;67(5):1152-62. doi: 10.1111/j.1365-2648.2010.05574.x. Epub 2011 Jan 27. PMID: 21272055.)

22	**Kangaroo Mother Care**	KMC is the care of preterm or LBW infants in continuous and prolonged (8–24 hours per day, for as many hours as possible) skin-to-skin contact recommended to be initiated immediately after birth, with support for exclusive breastfeeding or breast-milk feeding. An additional feature of KMC, when initiated in health-care facilities, is timely discharge from the neonatal intensive or special care unit to a lower level of care within the facility or at home, with continued skin-to-skin contact and close monitoring

		(World health Organization. Kangaroo mother care: a transformative innovation in health care. Global position paper. Geneva: WHO, Geneva; 2023. Available from: https://apps.who.int/iris/bitstream/handle/10665/367626/9789240072657-eng.pdf.
**23**	**Family-Centred Care**	Family centred care is a way of caring for children and their families within health services which ensures that care is planned around the whole family, not just individual child/person, and in which all the family members are recognized as care recipients.<br><br>(Shields L., Pratt J.&Hunter J. (2006) Family centred care: a review of qualitative studies. Journal of Clinical Nursing 15(10), 1317–1323.)
**24**	**Family-Centred Care**	Family-centered care is an approach to health care that is respectful of and responsive to individual family's needs and values<br><br>(Davidson JE, Aslakson RA, Long AC, Puntillo KA, Kross EK, Hart J, et al. Guidelines for Family-Centered Care in the Neonatal, Pediatric, and Adult ICU. Crit Care Med. 2017 Jan 1;45(1):103–28).
**25**	**Family-Centred Care**	Family centered care is an approach to health care that is respectful of and responsive to individual families' needs and values. The eight principles for patient centred and family-centred care for newborns in a neonatal intensive care unit are: parental access with no limitation due to staff shift or medical rounds, psychological support for parents, pain management, a supportive environment, parental support, skin-to-skin contact, support for breastfeeding and lactation and protection of sleep.<br><br>(WHO. Standards for improving the quality of care for small and sick newborns in health facilities. Geneva 2020. Available from https://iris.who.int/bitstream/handle/10665/353738/9789240043930-eng.pdf)
**26**	**Family-Centred Care**	FCC is defined as a philosophy of care that recognizes and respects the pivotal role which the family takes in the lives of children with special health needs. The major goal of this philosophy is to support families in their natural caregiving roles by building on unique individual and family strengths. This approach promotes the family as a partner with the health care of their child.<br><br>(Shelton T.L., Jeppson E.S. & Johnson B.S. (1987) Family-centered Care for Children with Special Health Care Needs. Association for the Care of Children's Health, Washington, DC.).
**27**	**Family-Centred Care**	Family-Centred Care (FCC) is an approach to the planning, delivery and evaluation of health care that is grounded in mutually beneficial partnership among health care providers, patients, and families. It redefines the relationships in health care by placing an emphasis on collaborating with people of all ages, at all levels of care, and in all health care settings. In patient- and family-centered care, patients and families define their "family" and determine how they will participate in care and decision-making. A key goal is to promote the health and well-being of individuals and families and to maintain their control. This perspective is based on the recognition that patients and families are essential allies for quality and safety—not only in direct care interactions, but also in quality improvement, safety initiatives, education of health professionals, research, facility design, and policy development.<br>Patient- and family-centered care leads to better health outcomes, improved patient and family experience of care, better clinician and staff satisfaction, and wiser allocation of resources.<br><br>(Institute for patient and family centered care - IPFCC. Patient and family centered care [Internet]. [cited 2023 Oct 22]. Available from: https://www.ipfcc.org/about/pfcc.html)
**28**	**Patient and family-centered care**	Patient- and family-centered care is an innovative approach to the planning, delivery, and evaluation of health care that is grounded in a mutually beneficial partnership among patients, families, and providers that recognizes the importance of the family in the patient's life.<br><br>(American Academy of Pediatrics, Committee on Hospital Care; Institute for Patient- and Family-Centered Care. Patient- and family-centered care and the pediatrician's role. Pediatrics. 2012;129(2):394–4040)

29	**Individualized Family-Centered Development Care**	Developmental care for infants is an approach that individualizes care by observing and interpreting the infant's behavior and thus modifying the environment and caregiving to meet the developmentally appropriate expectations of the infant's brain. Developmental care also should be individualized to meet the needs of families regardless of racial, ethnic, cultural, religious, socioeconomic, or language backgrounds. Developmental care in the complex congenital heart disease population incorporates a unique understanding of complex congenital heart disease and its physiological, mental, behavioral, and emotional effects on infants and their families. Developmental care is an overarching term that incorporates a constellation of interventions that can be integrated across the continuum of care.<br><br>(Lisanti AJ, Uzark KC, Harrison TM, Peterson JK, Butler SC, Miller TA, et al. Developmental Care for Hospitalized Infants With Complex Congenital Heart Disease: A Science Advisory From the American Heart Association. J Am Heart Assoc. 2023 Feb 7;12(3).)
30	**Infant and Family-Centered Development Care**	"…an individualized approach based on the infant's behavior with the parent acting as a primary team member."<br><br>(Browne JV, Jaeger CB, Kenner C; Gravens Consensus Committee on Infant and Family Centered Developmental Care. Executive summary: standards, competencies, and recommended best practices for infant- and family-centered developmental care in the intensive care unit. J Perinatol. 2020 Sep;40(Suppl 1):5-10. doi: 10.1038/s41372-020-0767-1. PMID: 32859958.)<br><br>*In accessory document pag 78:*<br><br>Infant family centered developmental care (IFCDC) = a process blending the natural unified growth of biologic and cognitive system organization and regulation of an infant with the continuous coordinated support of parent(s), family, and interprofessional health team.
31	**Infant and Family-Centered Development Care**	Infant and family-centred developmental care (IFCDC) is a descriptive term for a framework of newborn care that incorporates the theories and concepts of neurodevelopment, neuro-behaviour, parent-infant interaction, parental involvement, breastfeeding promotion, environmental adaptation, and change of hospital systems. The core pillars of IFCDC are: sensitive care based on infant behavioural communication and cues gives the infant a voice (1,2) and is beneficial for brain growth (5), parent engagement supports parental wellbeing and infant development (6–10), and customised adaptations of the NICU environment and hospital system as a whole.<br><br>(EFCNI. European standards of care for Newborn Health: Infant-& family-centered development care European foundation for the care of newborn infants. 2018 [Internet]. 2018. Available from: https://www.efcni.org/wp-content/uploads/2018/11/2018_11_16_ESCNH_Report_final.pdf)
32	**Development al care**	Developmental care refers to a range of strategies designed to modify the neonatal unit environment and modes of care to reduce the stressors on the developing brain.<br><br>(Griffiths, N., et al. (2019). "Individualised developmental care for babies and parents in the NICU: Evidence-based best practice guideline recommendations." Early Hum Dev **139**: 104840.)
33	**Development al care**	Developmental care is an evolving process where individualized caregiving interventions adjust to the developmental needs of an individual (in the present context, the preterm infant) communicated during interactions.<br><br>(Aita, M. and L. Snider (2003). "The art of developmental care in the NICU: a concept analysis." J Adv Nurs **41**(3): 223-232.)
34	**Newborn individualized development al care and assessment**	NIDCAP is an individualized developmental approach to support and care based on reading each preterm infant's behavioral cues, and on formulating a care plan, which enhances and builds upon the infant's strengths, and supports the infant in areas of sensitivity and vulnerability. The goal is the improvement of long-term child and family outcome. The framework applies throughout the infant's delivery process and admission

	**program (NIDCAP)**	to the NICU, and continues throughout the infant's hospital stay, the transition home, and the first few months at home. The comprehensive approach of NIDCAP, in which KMC plays an integral role, was created in an effort to decrease the discrepancy between the immature human brain's expectation for the all-embracing womb environment and the actual experience of a typical NICU. Thus, the goal of the NIDCAP relationship-based approach is to provide individualized, developmentally supportive, family centered care, which includes KMC, to each prematurely born infant and family in order to support their joint realization of optimal health and development.<br>NIDCAP is based on the following four assumptions: (1st) Detailed observations of infant behavior during daily care giving interactions provide an important basis for recommendations in how best to minimize stress and optimize an infant's development. (2nd) Parents and their closest supporters, often family members or friends, provide the optimal co-regulatory support and literal twenty-four-hour bed for the immature infant. (3rd) Care giving NICU staff benefits from supportive education in implementing the often challenging procedures necessary (e.g. suctioning, extubation, line placements, etc) as well as regularly available emotional support to process their complex feelings and self-doubt about having to give pain while simultaneously understanding the personhood of infant and parent who must trust and rely upon them. (4th) Resultant re-envisioning of care will lead to better outcome in infant medical well-being and neurobehavioral functioning, in parent well-being and functioning, and in staff professional and personal development.<br><br>(Als H, B. McAnulty G. The Newborn Individualized Developmental Care and Assessment Program (NIDCAP) with Kangaroo Mother Care (KMC): Comprehensive Care for Preterm Infants. Curr Womens Health Rev. 2011 Jul 11;7(3):288–301.)
**35**	**Universe of developmental care (UDC)**	The UDC model defines developmental care as a patient and family-centered process in which caregivers interact with the infant and the NICU environment at the level of the shared care surface to implement an individualized plan of care. Core measures of DSC include protected sleep, pain and stress assessment and management, developmental activities of daily living, family-centered care, and the healing environment.<br><br>(Gibbins S, Hoath S, Coughlin M, Gibbins A, Franck L. The universe of developmental care: a new conceptual model for application in the neonatal intensive care unit. Adv Neonatal Care. 2008 June;8(3):141-7.)
**36**	**Family integrated care (FICare)**	Family integrated care (FICare) is a transformative model of neonatal care which supports and mentors parents as primary care-givers for their infant in the neonatal intensive care unit (NICU). FICare seeks to directly address the negative impacts of the NICU environment on infants and their families and puts families at the centre of neonatal care delivery.<br>FICare comprises a comprehensive framework to implement a family centered care philosophy by bringing parents, medical and nursing staff together to develop a collaborative program of education and support; combining the strategies to support parent-infant interactions and highlighting the need for administration and infrastructure support. FICare promotes parental involvement and takes this to a new level by actively supporting parents as primary caregivers and equal partners in the care team. FICare uses a strength-based approach that focuses on the promotion of parent engagement and the development of parent self-efficacy. It shifts the paradigm of care so that neonatal staff provide education, mentorship, and coaching that focuses on the parent-infant dyad, where parents and infants are considered mutually dependent. The practical model of FICare developed by O'Brien et al in Toronto supports parents in building core constructs of self-efficacy (performance accomplishments, vicarious experiences, verbal encouragement, emotional support) through the use of four pillars.<br><br>(Waddington C, van Veenendaal NR, O'Brien K, Patel N. Family integrated care: Supporting parents as primary caregivers in the neonatal intensive care unit. Vol. 5, Pediatric Investigation. John Wiley and Sons Inc; 2021. p. 148–54.).
**37**	**Nurturing care**	Nurturing care refers to a stable environment that is sensitive to children's health and nutritional needs, with prot from threats, opportunities for early learning, and interactions that are responsive, emotionally supportive and developmentally stimulating. Nurturing care comprises five interrelated and indivisible components: good health, adequate nutrition, safety and security, responsive caregiving and opportunities for early learning

		(Black, M. M., et al. (2021). "The principles of Nurturing Care promote human capital and mitigate adversities from preconception through adolescence." BMJ Glob Health 6(4).
**38**	**Mother-Newborn Couplet Care**	Mother-Newborn Couplet Care is a concept where the care of the ill or prematurely born infant is provided, coupled with the care of the newly delivered mother in the same ward and uninterrupted from the birth of the child to discharge. The maternal care includes not only postpartum care, but also medical care for mothers with more complex medical needs such as caesarean section, pre-eclampsia, hypertension, infections diabetes<br><br>(Klemming S, Lilliesköld S, Westrup B. Mother-Newborn Couplet Care from theory to practice to ensure zero separation for all newborns. Acta Paediatr. 2021 Nov;110(11):2951-2957. doi: 10.1111/apa.15997. Epub 2021 Jul 1. PMID: 34146345.)
**39**	**Family-centered developmental care**	Family-centered developmental care (FCDC) takes family-centered care one step further by involving the family as an essential contributor to the provision of individualized, developmentally supportive care of their baby. FCDC provides the strong supportive foundation families in the neonatal intensive care unit (NICU) need to optimize the lifelong relationship between themselves and their babies, as well as to optimize the baby’s physical, cognitive and psychosocial development.<br><br>(Craig J, Glick C, Phillips R, Hall S, Smith J, Browne J. Recommendations for involving the family in developmental care of the NICU baby. J Perinatol 2015; 35:S5–S8)
**40**	**Neurodevelopmental care**	Neurodevelopmental care is a broad term applied to physician–nursing practices, physical environmental elements and family involvement philosophies that may favorably impact the neurodevelopment of the premature newborn. It has included promotion of positioning strategies, gentle touch, modulation of light and sound exposure, increased parental involvement, as well as an emphasis on the need to preserve sleep.<br><br>(Liu WF, Laudert S, Perkins B, Macmillan-York E, Martin S, Graven S, et al. The development of potentially better practices to support the neurodevelopment of infants in the NICU. J Perinatol. 2007;27(Suppl 2):S48-74)

Appendix 3: Identified categories of interventions for each model of care

This list presents the analysis of each model of care.

For each model of care, categories of interventions are listed and organised into macro-categories, using the following color coding.

Individualised neonatal healthcare
Organisation of care and human resources, policies
Physical resources
Health professionals capacity strenghtening and support
Family empowerment and support

1) Shuman CJ, et al. Integrating Neonatal Intensive Care Into a Family Birth Center: Describing the Integrated NICU (I-NIC). J Perinat Neonatal Nurs. 2023 Sep 28. doi: 10.1097/JPN.0000000000000759.

Family centred care practices (not otherwise specified)
Integrating maternal and newborn care
Enviromental redisign

2) Levin A. The Mother-Infant unit at Tallinn Children's Hospital, Estonia: a truly baby-friendly unit. Birth. 1994;21(1):39-44.

Breastfeeding promotion

Parental education

Parental partecipation to care

Hygiene promotion

3) Kapito EM, et al. The H-HOPE behavioral intervention plus Kangaroo Mother Care increases mother-preterm infant responsivity in Malawi: a prospective cohort comparison. BMC Pediatr. 2023 Apr 21;23(1):187. doi: 10.1186/s12887-023-04015-z.

KMC

Parents + (read/interpret/respond to infant's cues)

4) Lisanti AJ, et al. Developmental Care for Hospitalized Infants With Complex Congenital Heart Disease: A Science Advisory From the American Heart Association. J Am Heart Assoc. 2023 Feb 7;12(3).

Developmentally supportive feeding (and breastfeeding)
Nutrition and growth
Developmental and motor support (i.e. position, massage, strecthing)
Pain, sedation and withdrawal assesment, prevention and management
Interdisciplinary developmental care rounds
Discharge readineess and transition to home/community plans
Parental engagement and caregiving assessment
Parental mental health and psychosocial needs assessment

5) Chellani H, et al. Mother-Newborn Care Unit (MNCU) Experience in India: A Paradigm Shift in Care of Small and Sick Newborns. Indian J Pediatr. 2022 May;89(5):484-489. doi: 10.1007/s12098-022-04145-9. Epub 2022 Mar 4. PMID: 35244878; PMCID: PMC8895087.

Integrating maternal and newborn care
Enviromental redisign
Mother as caregiver not as mere visitor

6) de Salaberry J, et al. Journey to mother baby care: Implementation of a combined care/couplet model in a Level 2 neonatal intensive care unit. Birth Defects Res. 2019 Sep 1;111(15):1060-1072. doi: 10.1002/bdr2.1524.

Integration maternal and newborn care ("coordination of care")

Re-Education of staff with cross-training

Parental engagement with parents as primary caregiver

Peer-to-peer education and support

7) Hall SL, et al. The neonatal intensive parenting unit: an introduction. J Perinatol. 2017 Dec;37(12):1259-1264. doi: 10.1038/jp.2017.108.

Interdisciplinary collaboration

Palliative and bereavement care

Post-discharge follow up

Mental health professionals availability

Environmental changes (equipment, amenities) also for staff

Staff support

Staff education

Peer support and family support

Partnership with parents

Communication

8) Klemming S, et al. Mother-Newborn Couplet Care from theory to practice to ensure zero separation for all newborns. Acta Paediatr. 2021 Nov;110(11):2951-2957. doi: 10.1111/apa.15997. Epub 2021 Jul 1. PMID: 34146345.

Close collaboration between Obstetrics and Neonatal/Paediatric services

Unit design

Equipment adaptation

Adequate training of medical/nursing staff

9) Welch MG, et al. Family nurture intervention (FNI): methods and treatment protocol of a randomized controlled trial in the NICU. BMC Pediatr. 2012;12:14.

Supervision of "nurture specialists" nurses

Family support sessions

**10)** Maria, A., et al. (2021). "Nurturing Beyond the Womb- Early Intervention Practices in Newborn Care Unit." Indian Pediatr 58 Suppl 1: S53-s59.
Supportive positioning and handling
Tactile interventions
Vestibular interventions
Olfactory gustatory interventions
Kinesthetic interventions
Visual interventions
Reducing stress and pain
Protecting and promoting sleep
Nutrition
Skin care
Partnering with parents and families
Shift from provider centred to parents/provider shared model
Basic facilities for mothers/attendances (sleeping quarters/bathroom)
Peer-to-peer support
Parental training in clinical tasks
Respectful communication
Higyene training

**11)** Schuetz Haemmerli, N., et al. (2021). "Interprofessional Collaboration in a New Model of Transitional Care for Families with Preterm Infants- The Health Care Professional's Perspective." J Multidiscip Healthc 14: 897-908.
Physical therapy
Interprofessional roundtable discussions with parents and multidisciplinary team
Advanced practice nurse support
Lactation consultant
Psychological support
Social Worker support

**12)** Aita, M., et al. (2021). "Nurturing and quiet intervention (NeuroN-QI) on preterm infants' neurodevelopment and maternal stress and anxiety: A pilot randomized clinical trial protocol." J Adv Nurs 77(7): 3192-3203.
Skin to skin contact
Olfactory stimulation with breastmilk
Quiet period with reduced light and noise levels

13) Mhango P, et al. Implementing the Family-Led Care model for preterm and low birth weight newborns in Malawi: Experience of healthcare workers. Afr J Prim Health Care Fam Med. 2020 Aug 17;12(1):e1-e11. doi: 10.4102/phcfm.v12i1.2266

Quality improvement
Strengthening referral system and follow up care
Community sensitisation: peer-to-peer support. Volunteers and community health workers
Community engagement with take home materials for families
Improvement of equipment/supplies/infrastructure
Training and capacity building for health care cadres
Provides newborn monitoring forms for both staff/family
Orientation in family led-care: Use of pictorial counselling flipbook

14) Erdei C, et al. The Growth and Development Unit. A proposed approach for enhancing infant neurodevelopment and family-centered care in the Neonatal Intensive Care Unit. J Perinatol. 2019 Dec;39(12):1684-1687. doi: 10.1

Enviromental enrichement: multisensory + skin-to-skin + breastfeeding
Enhanced parental mental health strategy
GDU transdisciplinary team
Shared space for positive experiences
Family-led rounds
Increased parental involvement in care-giving and decision-making Informal peer-to-peer support
Parents education programs
Family financial and social support
Peer-to-peer support (parent-liasion partners with GDU)

15) Als H, B. McAnulty G. The Newborn Individualized Developmental Care and Assessment Program (NIDCAP) with Kangaroo Mother Care (KMC): Comprehensive Care for Preterm Infants. Curr Womens Health Rev. 2011 Jul 11;7(3):288–301.

Modification of external stimuli
Positioning and handling of infant
Detailed observation of infant behavior during daily care giving interactions
Clustering and coordination of nursery care activities

Staff benefits from supportive education in implementing the procedures (intubation/line placement)
Staff benefits from regularly available emotional support
Parents provide the optimal co-regulatory support

16) Staniszewska, S., et al. (2012). "The POPPY study: developing a model of family-centred care for neonatal units." Worldviews Evid Based Nurs 9(4): 243-255.

Overnight stay for parents
Post-discharge follow up with experienced staff (neonatal nurse)
Discharge planning with parental collaboration and clear communication
Prenatal consultation
Unrestricted regular contact with baby
Introduction to unit, staff, policies, patterns and routines
building parent confidence based on developing skills and knowledge
Parent-friendly information about unit policies
Support for parent/infant interaction and recognition of infant cues
Emotional support
Support schemes/peer support to learn from experience of other parents
Importance of parents having complete care resposability before discharge
Post-discharge support from health visitor
Access to support groups or community groups
Support programmes
Parent participation in ward rounds

17) Pricoco, R., et al. (2023). "Impact of a family-centred clinical care programme on short-term outcomes of very low-birth weight infants." Acta Paediatr 112(11): 2368-2377.

Discharge planning and post-discharge management (case managers)
Breastfeeding support with Lactation Consultant
Establisment of guiding principles, standard operating procedures, process description
Establishment of case managers
Rooming-in and 24 hr access for parents
Management team: multidisciplinary
Prenatal consultations
Change in infrastructure, reduction of noise and light. Spaces for privacy
Staff education
Specific educational/training sessions for small groups of parents
Parents competency programs
Parental empowerment program- parents as primary caregiver
Information material for parents
Psychological support

18) Patel, N., et al. (2018). "Family Integrated Care: changing the culture in the neonatal unit." Arch Dis Child 103(5): 415-419.

Staff education
Parent education
Peer-to-peer support
Communication

19) Waddington C, et al. Family integrated care: Supporting parents as primary caregivers in the neonatal intensive care unit. Vol. 5, Pediatric Investigation. John Wiley and Sons Inc; 2021. p. 148–54.

Design and implementation of all key components in partnership with families

A supportive environment (dedicated space for parents, physial support, foster connection between parent/infant)

Health-care provider education (engagement, Gap analysis, programs, mentorship, professional validation)

Parent psychoeducational support (Peer-to-peer, Professional support, Verbal encouragement/mentorship at bedside)
Parent education (Vicarious experiences, development of skills on infant care and medical needs, advocacy for developmental care)
Communication (parent participation in daily rounds, parent engagement in developing care plan)

20) Banerjee J, et al. Improving infant outcomes through implementation of a family integrated care bundle including a parent supporting mobile application. Arch Dis Child Fetal Neonatal Ed. 2020;105(2):172-7.

One-to-one support in lactaction and feeding

Environmental changes for parent support (optimised facilities and access)

Staff support and training

Parents presenting during wardrounds
Parent support (pyschologists and dedicated app)
Parent education (one-to-one and weekly small groups)

21) Murphy, M., et al. (2021). "Effectiveness of Alberta Family-Integrated Care on Neonatal Outcomes: A Cluster Randomized Controlled Trial." J Clin Med 10(24).

Parent support from professionals and family mentors (peer-to-peer)
Parent education, supported by technology and defined learning pathways Relational Communication

22) Altimier, L. and R. Phillips (2016). "The Neonatal Integrative Developmental Care Model: Advanced Clinical Applications of the Seven Core Measures for Neuroprotective Family-centered Developmental Care." Newborn and Infant Nursing Reviews 16(4): 230-244.

Healing environment (touch, smell, taste, sound, light)
Protecting skin
Positioning and handling
Safeguarding sleep
Minimizing stress and pain
Optimizing nutrition

Partenering with families

Training program for staff

Skin-to skin

23) Pineda R, et al. Supporting and Enhancing NICU Sensory Experiences (SENSE), 2nd Edition: An Update on Developmentally Appropriate Interventions for Preterm Infants. Children (Basel, Switzerland), 10(6), 961. https://doi.org/10.3390/children10060961

Tactile interventions (Includes skin to skin)
Kinesthetic interventions
Auditory interventions
Olfactory interventions
Visual interventions

Staff education (user manual + QI process)

Parents education (use of technology included)

24) McAlinden, B., et al. (2024). "'Baby Liberation'- Developing and implementing an individualised, developmentally-supportive care bundle to critically unwell infants in an Australian Paediatric Intensive Care Unit." Early Hum Dev 190: 105944.

Minimising pain and stress
Spontaneous awakening and breathing
Delirium and safeguarding sleep
Early mobility and positioning
Good nutrition

Family engagement and humanism (includes skin to skin)

25) Landsem IP, Handegård BH, Tunby J, Ulvund SE, Rønning JA. Early intervention program reduces stress in parents of preterms during childhood, a randomized controlled trial. Trials. 2014;15:387

Restructuring environment

Parental sensitivity training

Parental participation to care

Home evaluation and training

26) Czynski, A. J., et al. (2022). "The Mother Baby Comfort Care Pathway: The Development of a Rooming-In-Based Perinatal Palliative Care Program." Advances in neonatal care : official journal of the National Association of Neonatal Nurses 22(2): 119-124.

Rooming in

Coordination with multiple healthcare providers

Family support
Communication with family

27) WHO, (2023). "Global position paper. Kangaroo mother care: a transformative innovation in health care." Geneva.

Brestfeedung
Skin to skin
Early discharge
Follow up

28) Melnyk BM, Feinstein NF. Reducing hospital expenditures with the COPE (Creating Opportunities for Parent Empowerment) program for parents and premature infants: an analysis of direct healthcare neonatal intensive care unit costs and savings. Nurs Adm Q. 2009;33(1):32-7.

Parents performing activities to foster infants' development

Educational program
